# A Preliminary Study of Viral Metagenomics of French Bat Species in Contact with Humans: Identification of New Mammalian Viruses

**DOI:** 10.1371/journal.pone.0087194

**Published:** 2014-01-29

**Authors:** Laurent Dacheux, Minerva Cervantes-Gonzalez, Ghislaine Guigon, Jean-Michel Thiberge, Mathias Vandenbogaert, Corinne Maufrais, Valérie Caro, Hervé Bourhy

**Affiliations:** 1 Institut Pasteur, Lyssavirus Dynamics and Host Adaptation Unit, National Reference Centre for Rabies, WHO Reference Center for Reference and Research on Rabies, Paris, France; 2 Institut Pasteur, Genotyping of Pathogens and Public Health Platform, Paris, France; 3 Institut Pasteur, Computer Center for Biology, Paris, France; The University of Hong Kong, Hong Kong

## Abstract

The prediction of viral zoonosis epidemics has become a major public health issue. A profound understanding of the viral population in key animal species acting as reservoirs represents an important step towards this goal. Bats harbor diverse viruses, some of which are of particular interest because they cause severe human diseases. However, little is known about the diversity of the global population of viruses found in bats (virome). We determined the viral diversity of five different French insectivorous bat species (nine specimens in total) in close contact with humans. Sequence-independent amplification, high-throughput sequencing with Illumina technology and a dedicated bioinformatics analysis pipeline were used on pooled tissues (brain, liver and lungs). Comparisons of the sequences of contigs and unassembled reads provided a global taxonomic distribution of virus-related sequences for each sample, highlighting differences both within and between bat species. Many viral families were present in these viromes, including viruses known to infect bacteria, plants/fungi, insects or vertebrates, the most relevant being those infecting mammals (*Retroviridae*, *Herpesviridae*, *Bunyaviridae*, *Poxviridae*, *Flaviviridae*, *Reoviridae*, *Bornaviridae, Picobirnaviridae*). In particular, we detected several new mammalian viruses, including rotaviruses, gammaretroviruses, bornaviruses and bunyaviruses with the identification of the first bat nairovirus. These observations demonstrate that bats naturally harbor viruses from many different families, most of which infect mammals. They may therefore constitute a major reservoir of viral diversity that should be analyzed carefully, to determine the role played by bats in the spread of zoonotic viral infections.

## Introduction

Bats belong to the order Chiroptera, one of the most abundant, diverse and widely geographically dispersed groups of mammals. This order includes about 1,200 species, accounting for almost 25% of class Mammalia. Interest in these animals is increasing, as they are thought to play an essential role in ecosystem ecology, a domain that has only recently begun to be studied. Bats are highly diverse in terms of their anatomy and lifestyles. Most feed on insects and other arthropods, but some species feed on vertebrates, fish, blood, fruit, nectar or pollen. The order Chiroptera is divided into two suborders: Yinpterochiroptera (formerly Megachiroptera, corresponding to megabats) and Yangochiroptera (formerly Microchiroptera, corresponding to microbats). Bats occur on all continents other than Antarctica. In total, 36 bat species have been identified in Europe. All are insectivorous microbats and they are classified into four different families: *Rhinolophidae*, *Vespertilionidae*, *Molossidae* and *Miniopteridae*. Thirty-four of these bat species, from all four families, are found in France (excluding French overseas territories).

In recent years, bats have been the focus of particular interest not just because of their ecological interest, but also for public health reasons, as they are considered to play a major role in the emergence and transmission of zoonotic viral agents. Indeed, these animals act as a natural reservoir for many viruses [1]–[6], some of which, such as henipaviruses [7]–[9], severe acute respiratory syndrome coronavirus [10], Ebola and Marburg virus [11], [12] or lyssaviruses [13]–[15], cause serious human diseases. Studies based on PCR detection followed by classical sequencing approaches have demonstrated that bats naturally harbor many different viruses and have provided evidence that viral epidemics may result from the cross-species transmission of viruses from this reservoir to humans or other animals. The crossing of the species barrier by viruses is also facilitated by the expansion of the human population and the destruction of natural bat habitats, bringing bats into ever-closer contact with humans and other animals. It has been suggested that bats may harbor many other unknown, potentially epidemic viruses. However, the diversity of the global population of viruses found in bats (virome) remained little investigated until recently. The development of sequence-independent amplification approaches and their application, followed by high-throughput sequencing, to bat samples, including guano and saliva in particular, has led to the discovery of considerable viral diversity in bat species, through the identification of a large, growing number of viral species [16]–[22]. However, most known bat viruses were discovered in apparently healthy bats [19], [23]–[28] or were detected directly in guano samples collected from the floors of caves harboring colonies [16]–[18], [20]. To date no study has investigated the diversity of viruses in sick bats that have been in direct contact with humans. Analyses of the viromes of bats from this particularly epidemiological situation in the field, reflecting close contact with humans, constitute a crucial step in assessments of the risk of spill-over transmission, and in the prediction and prevention of viral epidemics.

We describe here the viral diversity found in nine French bat specimens from five different species with anthropophilic behavior. All these dead bats were sent to the national reference centre for rabies (NRC-Rabies) for testing, after they had come into direct contact with humans. Most had displayed abnormal behaviour before their deaths. The global taxonomic distribution of virus-related contig and read sequences was determined for each sample. It highlighted differences both within and between bat species. A large number of viral families were represented in the viromes obtained, but most of the viral sequences were from vertebrate viruses, particularly those preferentially infecting mammals. A significant proportion of virus-related contigs had low levels of sequence identity to known viral genomic or protein sequences, suggesting the presence of new, genetically diverse bat viruses.

## Materials and Methods

### Bat Specimens and Tissue Sample Collection

We selected nine French bat carcasses, all from insectivorous species, from the collection stored at −80°C at NRC-Rabies. All these carcasses were sent to the center in the framework of the French program for rabies surveillance set up by the national veterinary agency, which has a service related to health and animal welfare in each county. Depending on the epidemiological context, veterinary inspectors of this service authorized the shipment and then the subsequent analysis by the NRC-Rabies for diagnosis of animals at risk of rabies exposure in humans. The suspected animals are mainly brought directly by the owners or by patients to private veterinary clinics, which are in charge to transfer them to the local veterinary agencies where they are usually stored at +°4C before shipment. All specimens of bats in this study were found dead or died naturally after collection. These dead animals were sent by the local veterinary agencies between 2009 and 2010, following incidents of potential human exposure to rabies through bites, scratches, licking or hand manipulations, requiring the mandatory submission of the bat carcasses for rabies testing (according to French regulations defined by ministerial orders NOR N°MESP0220774A, SANP0424067A and SASP1017259A, and by article law N°223-36 of the rural and maritime fishing code, subsequently amended by decree NOR N°AGRG1100274D). All the bats studied tested negative for rabies by conventional techniques at the NRC-Rabies [29]. Autopsy was performed in a biosafety level 3 cabinet, with sterilized instruments, to prevent cross-contamination. For each specimen, we harvested three different organs – the lungs, liver and brain (when available) – to provide a representative selection of target organs for respiratory, digestive and nervous system infection, respectively. We used about 50 mg of each organ for RNA extraction from individuals. All the bats analyzed were adults. Specimen and sample information, including family, species, sex, location, date of collection and other epidemiological data, such as the type of contact with humans, is provided in Table 1. Bats were identified to species level on the basis of anatomical and morphological characters.

**Table 1:** Description of bat specimens and related epidemiological data.

		Bat specimens and tissue samples				Epidemiological data			
Sampleidentification	Code	Species	Common name	Sex	Organ^a^	Location^b^	Collectiondate	Type of humancontact	Otherinformation
b1	2009/01012	*Pipistrellus pipistrellus*	Common pipistrelle	Male	Br, li, lu	Moulins (Allier-03)	August, 2009	Hand manipulation	Found dead in a secondary school
b2	2009/00385	*Pipistrellus pipistrellus*	Common pipistrelle	Male	Br, li, lu	Paris (Paris-75)	April, 2009	Hand manipulation	Caught by a cat
b3	2009/00820	*Pipistrellus pipistrellus*	Common pipistrelle	Female	Br, li, lu	Bordeaux (Gironde-33)	July, 2009	Hand manipulation	Manipulation by a girl playing with the animal
b4	2009/00886	*Pipistrellus pipistrellus*	Common pipistrelle	Male	Br, li, lu	Courtry(Seine-et-Marne-77)	July, 2009	Hand manipulation	Found sick and died one day later
b5	2009/00865	*Hypsugo* *savii*	Savi’s pipistrelle	Male	Br, li, lu	Cannes la Bocca(Alpes-Martimes-06)	July, 2009	Bite, scratches	Caught by a cat
b6	2010/00513	*Myotis* *nattereri*	Natterer’s bat	Male	Br, li, lu	Latresne(Gironde-33)	June, 2010	Hand manipulation	Found dead in a garden where children were playing
b7	2010/00553	*Eptesicus* *serotinus*	Common serotine	Male	Br, li, lu	Sers (Charentes-16)	June, 2010	Hand manipulation	Collected in a sanctuary, then became sick and died one day later
b8	2009/01073	*Myotis* *mystacinus*	Whiskered bat	Male	Li, lu	Maule(Yvelines-78)	September, 2009	Bite	Found weak, died during the monitoring
b9	2009/00996	*Pipistrellus pipistrellus*	Common pipistrelle	Male	Li, lu	Ahun (Creuse-23)	August, 2009	Hand manipulation	Found dying

**Legend :**

a Br = brain, li = liver, lu = lungs.

b City and county with related county codification.

### Extraction of RNA and Sequence-independent Amplification

Tissue samples were disrupted and homogenized with plastic pistons using specific buffers including in the Allprep DNA RNA Mini kit (Qiagen) or in TriReagent (Euromedex) for liver and lungs or for brain, respectively. Total RNA was then extracted according to the manufacturer’s protocols. In addition, a final step of purification was performed for RNA extracted from brain using RNeasy kit (Qiagen). All RNA were eluted in 30 µl of RNase-free water and stored at −80°C until use. They were then subjected to reverse transcription followed by amplification with the whole-transcriptome amplification (WTA) protocol (QuantiTect Whole Transcriptome kit, Qiagen), as previously described [30]. The WTA products obtained from the various organs collected from a single bat specimen were pooled (mixing of equal volumes) before sequencing.

### High-throughput Sequencing

We sheared 1 µg of high-molecular weight double-stranded DNA with the Bioruptor® protocol, according to the manufacturer’s recommendations. The DNA fragments generated were about 350 bp long and were used to construct a genomic library with the TruSeq DNA sample prep kit V2 (Illumina), according to the manufacturer’s recommendations. Quadruplex pooled libraries were sequenced on an Illumina HiSeq-2000 platform to give 100 bp paired reads, with the TruSeq PE Cluster kit v3 and TruSeq SBS kit v3 (Illumina).

### Bioinformatics Analysis and Taxonomic Assignment

We used a bioinformatics workflow for metagenomic characterization from large sets of short sequence reads and their assemblies, such as those typically generated by high-throughput sequencers including the Illumina HiSeq 2000. In this study, we focused on the virome of bats. Data quality is very important for various downstream analyses, including sequence assembly in particular. We therefore applied a pre-processing workflow to ensure that high-quality reads were obtained. The following “cleaning” steps were applied to the raw reads: (1) reads with at least a given user-defined number of bases with Phred quality scores were selected (Sanger quality >20 and remaining length >26 nt); (2) a user-defined primer/adaptor sequence list was compared with the remaining reads, such that reads containing these primer/adaptor sequences were eliminated; (3) reads of less than a given length were removed; (4) homopolymer-containing reads were trimmed and (5) duplicated reads were identified and removed.

The most closely related host genome sequences available (in this study, *Myotis lucifugus* whole-genome shotgun sequencing project accession number AAPE00000000, version of September 2010) were scanned and discarded with SOAP2 mapper software [31]. All homology searches in the bioinformatics process were run on a high-performance computing cluster, using BLAST via an optimized, load-balancing job scheduler. A rapid, highly restrictive BLASTn homology search (with word size 40) against a non redundant nucleotide (nt) database (retrieved/updated on March 2012) was performed, to eliminate additional host reads and eukaryotic contaminants. A *de novo* assembly of the remaining reads was built with Velvet-optimiser version 2.2.0 [32] and CLC Genomics Workbench version 3 (CLC Bio, Cambridge, MA). BLASTn and BLASTx were used to check for sequence identity between the contigs generated with Velvet and CLC and the sequences in a non redundant nt database (E value ≤10e-3) and UniProt (E value ≤1000) maintained locally (retrieved/updated on March 2012), respectively. For each contig, the best hit from BLASTn searches and the five best hits from BLASTx searches were retrieved, respectively. Unassembled reads were also used for BLASTn homology searches (E value ≤10e-5) against the non redundant nt database, and the five best hits exhibiting the best scores were retrieved. Based on all these BLAST results (contigs and unassembled reads), a taxonomic report was prepared for each sample, using an optimized in-house distributed program, pTaxOptimizer, to retrieve taxonomic information from the NCBI taxonomy database (ftp://ftp.ncbi.nlm.nih.gov/pub/taxonomy/) or from the taxonomy field of GenBank database.

Larger viral contigs were obtained and their position in the genome determined, by pooling contig and read sequences for a given viral family for each sample, and assembling them with Sequencher 5.0 software (Gene Codes Corporation). We used BLASTn and BLASTx against the EMBL and UniProt databases, respectively, with an E value of 10000 for both. Taxonomic classification was then undertaken with the blast2taxoclass program (http://mobyle.pasteur.fr/cgi-bin/portal.py#forms::blast2taxoclass), and sequences assigned to viruses were then annotated with blast2genoclass (http://mobyle.pasteur.fr/cgi-bin/portal.py#forms::blast2genoclass), as previously described [33]. Classified contig sequences were extracted and analyzed further in phylogenetic studies or used for the design of specific primers.

### Molecular Detection of Specific Viruses

Specific PCR primers were designed and used to screen for the presence of mammalian (nairovirus, rotavirus, gammaretrovirus and bornavirus) and insect (dicistrovirus and nodavirus) viruses in each individual organ: the lungs, livers and brains (when available) collected from the nine bat specimens. The sequence-independent amplification products were used as the starting material. Primer sequences and PCR conditions are available on request. The amplified products were sequenced and used to determine the accuracy of the original contig sequences.

### Phylogenetic Analysis

Selected contig sequences or sequenced PCR products were used for phylogenetic analysis. Reference nucleotide or protein sequences from the various viral families studied were downloaded from the GenBank and UniProt databases, respectively. Multiple alignments were generated with ClustalX version 2.0 [34], [35] and used in BioEdit [34]. Amino-acid sequence alignments were checked by eye, manually refined if necessary and trimmed to match the genome regions corresponding to the contig sequences or the sequenced PCR products generated in this study. Phylogenetic analyses were carried out by the neighbor-joining method, with MEGA 4.0.2 [36] and a Poisson correction model for amino-acid sequences or a Kimura two-parameter model (transition-to-transversion ratio of 2.0) for nucleotide sequences, both with 1,000 bootstrap replicates. Trees were visualized with FigTree version 1.3.1 (available from http://tree.bio.ed.ac.uk/software/figtree). The GenBank or UniProt accession numbers of the viral sequences used in the phylogenetic analyses are indicated in the trees.

### Nucleotide Sequence Accession Numbers

The GenBank accession numbers for the sequences of the partial viral genomes (high-scoring segment pairs (HSPs) or sequenced PCR products) obtained during this study and used in the phylogenetic analysis are KF170221–KF170229. The data from Illumina sequencing have been deposited in the GenBank Sequence Reads Archive under accession numbers SAMN02437308–SAMN02437316.

## Results

### Global Analysis of High-throughput Sequencing Data

We generated about 20 million raw read sequences (each about 100 nt long) for each sample. About 68.5% of all reads were retained after quality trimming and duplicate removal processes, the proportion retained ranging from 97.8% (specimens b6, b8 and b9) to 30.5% (specimen b3) (Table 2). This proportion of retained reads was further decreased by the removal of host reads, to between 68.6% (specimen b7) and 10.5% (specimen b3), depending on the sample considered.

**Table 2:** Overview of read and contig sequences.

												Assembly data on filtered reads (%)										
			Number of reads remaining after filtering (%)									Velvet assembly						Clc assembly				
								Host reads removal				Contigs			Unassembled reads			Contigs			Unassembled reads	
Sample	Quantity of amplified dbDNA used (µg)	Total number of reads	Quality trimming		Duplicate removal			Mapping		Blast W40		Number	Mean length (nt)	Number. of viral contigs	Number	Mean length (nt)	Number of viral reads	Number of contigs	Mean length (nt)	Number of viral contigs	Number	Mean length (nt)
b1	5	12,899,799	12,645,028 (98)		7,023,394 (54.4)			6,038,080 (46.8)		4,204,267 (32.6)		6,751	306	11 (0.2)	3,552,001 (27.5)	79	2,199 (0.02)	36,190	382	555 (1.5)	1,889,481 (14.6)	86
b2	5	22,219,022	21,950,265 (98.8)		10,115,989 (45.5)			8,039,808 (36.2)		7,879,238 (35.5)		4,951	330	11 (0.2)	3,831,327 (17.2)	80	1,945 (0.01)	36,539	352	784 (2.1)	2,055,437 (9.2)	87
b3	2.5	30,302,694	29,851,514 (98.5)		9,238,554 (30.5)			7,389,992 (24.4)		3,169,933 (10.5)		5,324	340	26 (0.5)	2,687,511 (8.9)	70	3,435 (0.02)	10,965	555	84 (0.8)	1,210,940 (4)	72
b4	2.5	20,235,147	19,940,937 (98.5)		10,952,524 (54.1)			10,070,367 (49.8)		7,548,853 (37.3)		6,532	370	9 (0.1)	6,450,250 (31.9)	79	4,657 (0.02)	30,450	374	342 (1.1)	4,081,845 (20.2)	81
b5	2.5	10,421,980	10,248,486 (98.3)		4,265,414 (40.9)			3,669,459 (35.2)		2,340,303 (22.4)		1,376	235	0	2,050,634 (19.7)	76	645 (0.01)	6,001	314	79 (1.3)	1,443,245 (13.8)	81
b6	5	23,479,591	22,961,278 (97.8)		22,961,130 (97.8)			18,329,510 (78.1)		1,057,387 (42.8)		227	275	21 (9.2)	1,920,758 (8.2)	7	15,569 (0.07)	15,768	490	202 (1.3)	5,345,900 (22.8)	69
b7	5	15,298,971	14,929,686 (97.6)		14,929,647 (97.6)			12,630,582 (82.5)		10,490,399 (68.6)		3,575	216	6 (0.2)	8,320,883 (54.4)	66	12,382 (0.08)	15,214	403	216 (1.4)	6,664,323 (43.6)	68
b8	5	22,141,787	21,652,636 (97.8)		21,652,569 (97.8)			17,122,319 (77.3)		9,442,165 (42.6)		1,693	368	38 (2.2)	8,314,006 (37.5)	66	170,833 (0.8)	14,807	496	184 (1.2)	5,205,772 (23.5)	67
b9	5	19,876,848	19,437,257 (97.8)		19,437,195 (97.8)			15,342,225 (77.2)		10,842,780 (54.5)		25	341	1 (4)	10,384,671 (52.2)	67	43,742 (0.2)	13,586	576	106 (0.8)	5,922,754 (29.8)	68
Mean	-	19,652,871	19,290,787 (98.2)		13,397,380 (68.2)			10,959,149 (55.8)		7,330,592 (37.3)		3,384	309	14 (0.4)	5,279,116 (26.9)	66	28,379 (0.14)	16,126	438	284 (1.2)	3,757,744 (19.1)	75
Mean deviation	-	4,519,525	4,455,369		5,642,449			4,352,898		2,728,283		2,270	45	10	2,745,188	13	35,071	5,832	81	191	1,873,750	7

**Figure 1:**
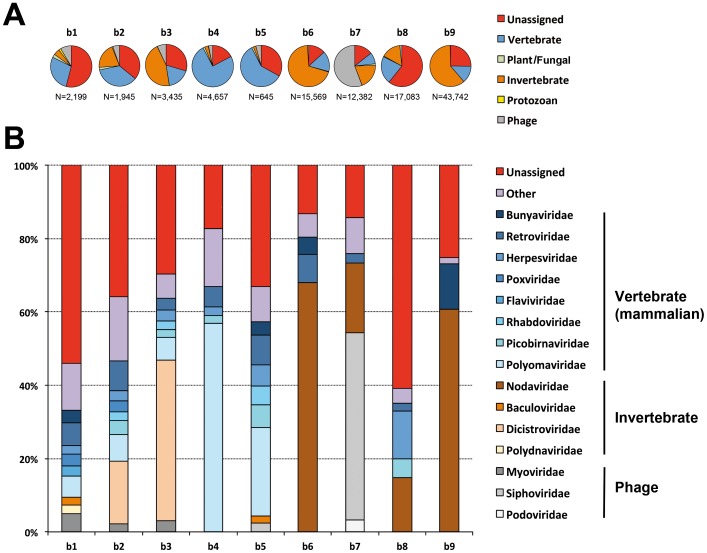
Distribution of unassembled read sequences after BLASTn analysis. (A) Percentage of sequences related to the main categories of existing viruses: vertebrate (blue), plant/fungal (green), invertebrate (brown), protozoan (yellow) viruses and bacteriophages (gray), and unassigned viral sequences (no taxonomic data concerning the family available, indicated in red). The total number of viral read sequences is indicated below each pie chart. (B) The percentage of sequences related to the most abundant viral families, indicated in the same colors for each main viral category as in (A): blue = vertebrate, brown = invertebrate, gray = phage. Viral families accounting for less than 2% of total sequences were pooled and represented as the “other” category (in purple), and read sequences with no available data concerning the taxonomic family were considered to be unassigned (in red).

Contig sequences were then generated by *de novo* assembly, using the Velvet and CLC Genomics Workbench programs, generating almost 3,400 contigs (mean length = 309 nt) and 16,100 contigs (mean length = 438 nt) per pool sample, respectively (Table 2). A first taxonomic assignment of these contig datasets was performed, on the basis of BLASTn (E value ≤10e-03) or BLASTx (E value ≤1000) analysis, after Velvet and CLC *de novo* assemblies, respectively. At this stage, the proportion of viral contigs was low, accounting for less than 2% of the total number of contigs (Table 2). These two types of contigs were pooled for each sample and analyzed for taxonomic classification (Fig. 1A and 1B). The limited number of contigs obtained with Velvet was offset by classifying unassembled reads in a similar manner after BLASTn analysis. Virus-related reads accounted for less than 0.2% of all raw read sequences, consistent with the findings of previous metagenomic studies in bats based on Illumina sequencing (Table 2) [17], [20], [22].

**Figure 2:**
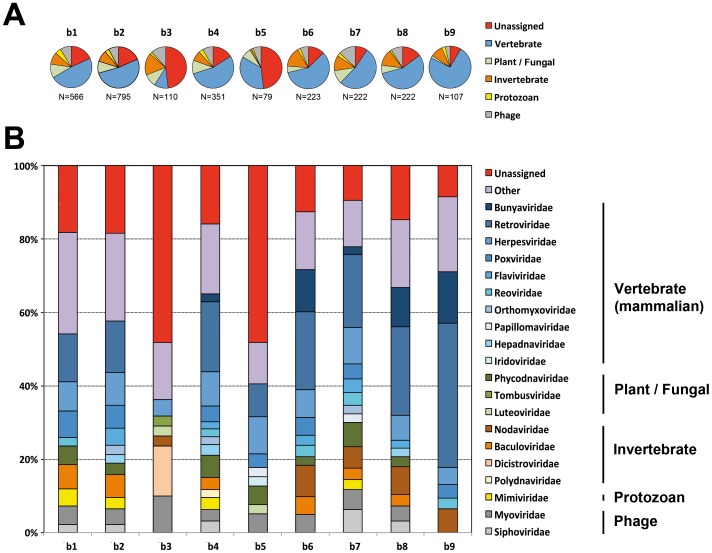
Distribution of contig sequences after BLASTx analysis. (A) Percentage of sequences related to the main categories of existing viruses: vertebrate (blue), plant/fungal (green), invertebrate (brown), protozoan (yellow) viruses and bacteriophages (gray), and unassigned viral sequences (no data available concerning the taxonomic family, indicated in red). The total number of viral contigs is indicated below each pie chart. (B) Percentage of sequences related to the most abundant viral families, indicated by the same color range for each of the main viral categories as in (A) : blue = vertebrate, green = plant/fungal, brown = invertebrate, yellow = protozoan, gray = phage. Viral families accounting for less than 2% of all sequences are pooled in the “other” category (in purple), and read sequences with no available data regarding the taxonomic family are considered to be unassigned (in red).

Viral composition differed both between and within bat species (Fig. 1A and 2A). The proportions of unassigned viral contig sequences and read sequences were 22% and 32% (mean values), respectively. Most contigs had low levels of nucleotide or amino-acid similarity to known viral sequences from the databases queried, suggesting that these sequences corresponded to previously unknown or genetically distant viruses, as reported in previous bat viral metagenomics studies [16]–[18], [20]–[22].

Sequences from vertebrate viruses, and from mammalian viruses in particular, predominated. The principal viral families identified included the *Bunyaviridae*, *Flaviviridae*, *Herpesviridae*, *Poxviridae*, *Reoviridae* and *Retroviridae*, some of which were also found in previous bat virome studies [16], [18], [20]–[22] (Fig. 1B and 2B).

Virus infecting invertebrates (mostly insects; 10% of contigs and 27% of reads, respectively, mean values), plants/fungi (8% and <2%, respectively) and protozoa (3% and <2%, respectively) or bacteriophages (8% and 10%, respectively), were also present, albeit at a lower frequency. Invertebrate virus-related contig sequences mostly related to the *Nodaviridae*, *Baculoviridae* and *Dicistroviridae* families, whereas the sequences related to viruses infecting plants were mostly associated with *Phycodnaviridae*, *Tombusviridae* or *Luteoviridae*, and those related to viruses infected protozoa were mostly associated with the family *Mimiviridae* (Fig. 1B and 2B). There was a large proportion of phage-related contigs and reads, which displayed similarities to sequences from myoviruses, podoviruses and siphoviruses.

### Identification of New Mammalian Viruses

We focused on the identification of a selected panel of new mammalian viruses, from the *Bunyaviridae, Reoviridae*, *Retroviridae* and *Bornaviridae*. This selection was based on the abundance of read and contig sequences corresponding to a given viral family, the length of the contig sequences obtained and the interest of the viral family identified in terms of the potential emergence of zoonoses and an absence or low frequency in bats.

#### Identification of a new nairovirus

**Figure 3:**
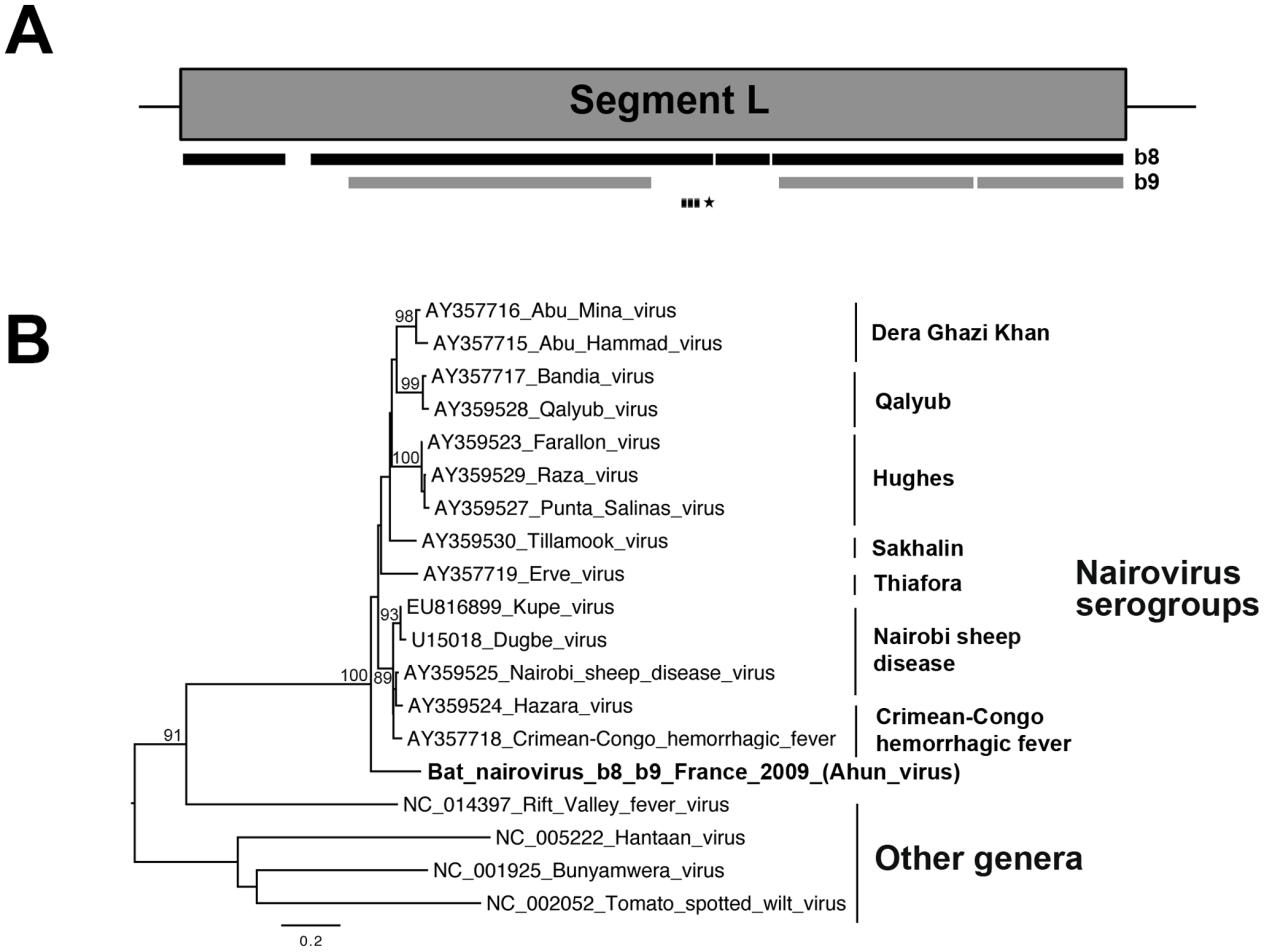
Phylogenetic analysis of the bat nairovirus-related sequences. (A) Schematic representation of the large (L) segment (almost 12,260 nt encoding the RNA-dependent RNA polymerase of almost 4,040 aa) from the genome of Dugbe virus (GenBank number U15018), with black and gray bars corresponding to the longest contig sequences (>300 nt) of the bat nairovirus (named Ahun nairovirus) identified in specimens b8 and b9, respectively. The genomic region amplified by PCR is represented by a dashed bar, and the sequence used for phylogenetic analysis is indicated with an asterisk. (B) Phylogenetic tree produced from the amino-acid alignment of the partial polymerase fragment (396-nt sequence, translated into a 132-aa sequence, aa positions 2317 to 2448 of the L protein of Dugbe virus, and aligned in accordance with previous studies [86], [87]). The name of the bat nairovirus is indicated in bold. The various serogroups of nairoviruses and prototype species of the other genera belonging to the family *Bunyaviridae* are indicated on the right of the tree. The scale bar indicates branch length, and bootstrap values ≥70% are shown next to the relevant nodes. The tree is midpoint-rooted for purposes of clarity only.

The genus *Nairovirus,* from the family *Bunyaviridae*, has seven species groups (or serogroups), including at least 34 predominantly tick-borne viruses [37], [38]. Like other members of this family, the viruses of this genus have a genome consisting of three segments of negative-sense, single-stranded RNA: the small (S), medium (M) and large (L) segments, encoding the nucleocapsid (N) protein, the glycoprotein precursor (GPC) generating the mature glycoproteins (Gn and Gc) on cleavage, and the viral polymerase (L), respectively [39], [40]. Multiple reads and several large contigs (up to almost 4,900 nt in length) from specimens b8 and b9 (liver and lung pools from *Myotis mystacinus* and *Pipistrellus pipistrellus*, respectively) strongly matched sequences from nairoviruses (Table 3). All three genome segments were covered (Fig. 3A and data not shown). For each segment, a comparison of overlapping nucleotide regions (up to 3,456 nt long) between b8 and b9 contig sequences showed complete nucleotide identity, indicating that the same nairovirus-related agent was present in those two bat species (data not shown). All individual bat tissue samples were screened by PCR for the presence of this virus, with a specific set of primers targeting a conserved domain of the polymerase sequence. Lung tissue samples from specimens b8 and b9 tested positive on the amplification and sequencing of the amplicons (Table 3). Phylogenetic analysis demonstrated that this bat-related nairovirus diverged considerably from all other nairoviruses identified to date (Fig. 3B). Thus, this virus, which we have named Ahun nairovirus, represents a putative new species of the genus *Nairovirus*, and the first bat nairovirus identified to date.

**Table 3:** Identification and distribution of sequences of vertebrate viruses of interest among the various bat specimens and tissue samples analyzed.

		Bat species, specimens and tissue samples^a^																								
		*Pipistrellus pipistrellus*														*Hypsugo savii*			*Myotis nattereri*			*Eptesicus serotinus*			*Myotis mystacinus*	
		b1			b2			b3			b4			b9		b5			b6			b7			b8	
Virus	Sequence detection^b^	Br	Li	Lu	Br	Li	Lu	Br	Li	Lu	Br	Li	Lu	Li	Lu	Br	Li	Lu	Br	Li	Lu	Br	Li	Lu	Li	Lu
Nairovirus	HTS	−c (tissue pool)			− (tissue pool)			− (tissue pool)			− (tissue pool)			+^c^ (tissue pool) *[S, M, L]*		− (tissue pool)			− (tissue pool)			− (tissue pool)			+ (tissue pool) *[S, M, L]*	
	PCR/Sanger	−	−	−	−	−	−	−	−	−	−	−	−	−	+ *[L]*	−	−	−	−	−	−	−	−	−	−	+ *[L]*
Rotavirus	HTS	− (tissue pool)			− (tissue pool)			− (tissue pool)			− (tissue pool)			− (tissue pool)		− (tissue pool)			− (tissue pool)			− (tissue pool)			+ (tissue pool) *[VP1–2, VP4, VP7]*	
	PCR/Sanger	−	−	−	−	−	−	−	−	−	−	−	−	−	−	−	−	−	−	−	−	−	−	−	−	+ *[VP1–2]*
Gammaretrovirus	HTS	− (tissue pool)			− (tissue pool)			− (tissue pool)			− (tissue pool)			− (tissue pool)		− (tissue pool)			− (tissue pool)			+ (tissue pool) *[env, pol]*			− (tissue pool)	
	PCR/Sanger	−	−	−	−	−	−	−	−	−	−	−	−	−	−	−	−	−	−	−	−	−	-	+ *[pol]*	−	−
Bornavirus	HTS	+ (tissue pool) *[L]*			− (tissue pool)			− (tissue pool)			− (tissue pool)			− (tissue pool)		− (tissue pool)			+ (tissue pool) *[L]*			− (tissue pool)			− (tissue pool)	
	PCR/Sanger	+ *[L]*	−	−	+ *[L]*	−	−	+ *[L]* d	−	+ *[L]*	+ *[L]*	−	−	+ *[L]* d	−	+ *[L]* d	−	+ *[L]*	+ *[L]*	−	+ *[L]* d	−	−	−	−	−
Adenovirus	HTS	− (tissue pool)			− (tissue pool)			− (tissue pool)			− (tissue pool)			− (tissue pool)		− (tissue pool)			**+** (tissue pool) *[multiple genes]*			− (tissue pool)			− (tissue pool)	
	PCR/Sanger	ND^e^	ND	ND	ND	ND	ND	ND	ND	ND	ND	ND	ND	ND	ND	ND	ND	ND	ND	ND	ND	ND	ND	ND	ND	ND
Picobirnavirus	HTS	+ (tissue pool) *[RdRp]*			− (tissue pool)			− (tissue pool)			+ (tissue pool) *[RdRp]*			+ (tissue pool) *[RdRp]*		+ (tissue pool) *[RdRp]*			− (tissue pool)			− (tissue pool)			+ (tissue pool) *[RdRp]*	
	PCR/Sanger	ND	ND	ND	ND	ND	ND	ND	ND	ND	ND	ND	ND	ND	ND	ND	ND	ND	ND	ND	ND	ND	ND	ND	ND	ND

**Legend :**

a Br = brain, Li = liver, Lu = lungs.

b Matching (HTS) or targeting (PCR/Sanger) viral genes (in brackets), with S = small, M = medium, L = large or polymerase, RdRp = RNA-dependent RNA polymerase.

c + = positive, - = negative.

d PCR products larger than expected.

e ND = not done.

#### Identification of a new bat rotavirus

**Figure 4:**
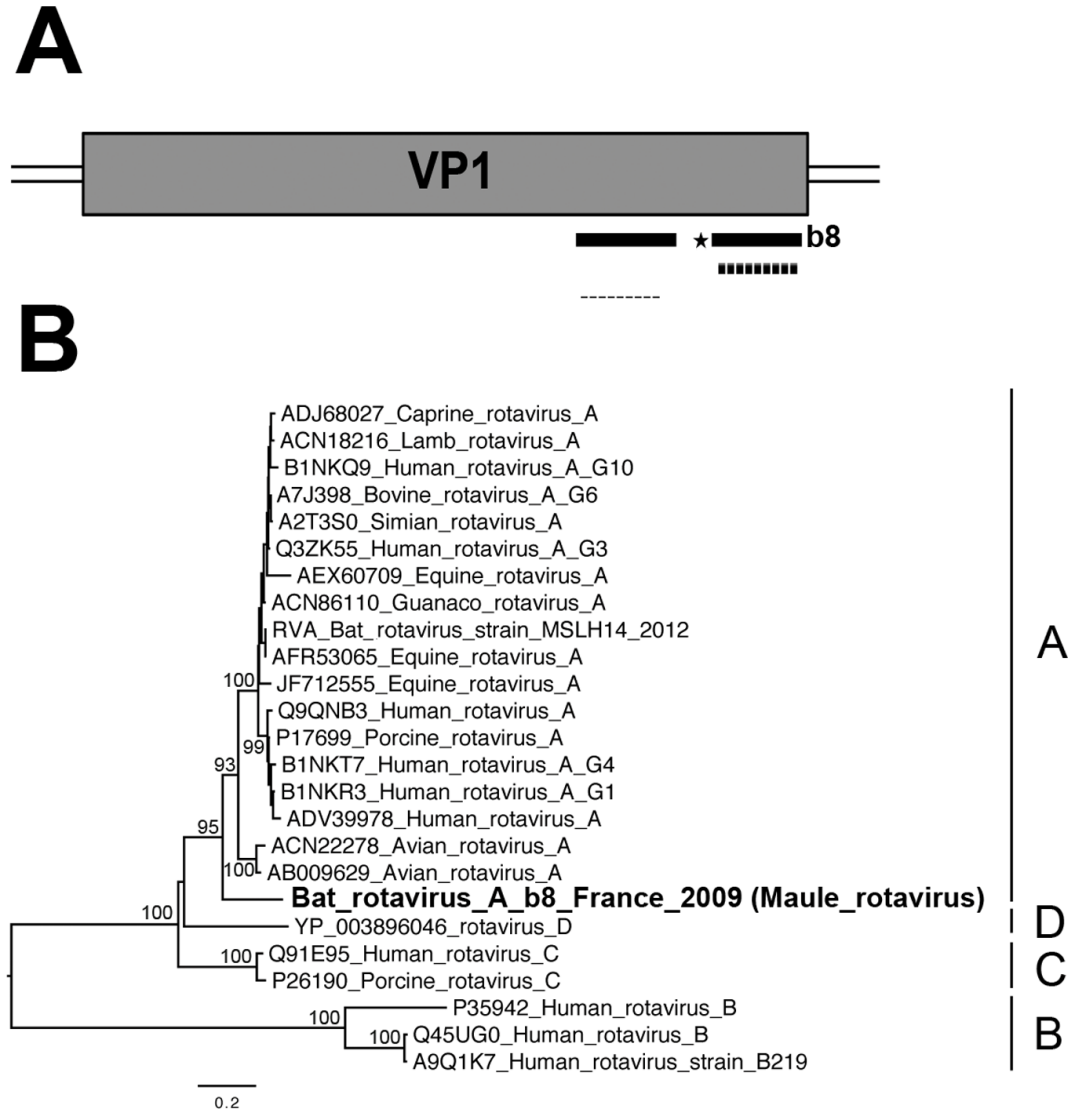
Phylogenetic analysis of VP1 bat rotavirus-related sequences. (A) Schematic representation of the VP1 segment (almost 3,300 nt encoding the RNA-dependent RNA polymerase of almost 1,090 aa) of the genome of the lamb rotavirus strain Lamb-NT (GenBank number FJ031024), with black bars corresponding to the longest contig sequences (>300 nt) of the bat rotavirus (named Maule rotavirus) identified in specimen b8 (*Myotis mystacinus*). The genomic region amplified by PCR is represented by a dashed bar, and the sequence used for phylogenetic analysis is indicated with an asterisk. B) Phylogenetic tree produced from the amino-acid alignment based on the partial VP1 sequence (119 aa, positions 964 to 1082 of the VP1 protein of lamb rotavirus strain Lamb-NT) translated from one of the longest HSPs. The bat rotavirus-related sequence is indicated in bold within the various rotavirus groups. The scale bar indicates branch length, and bootstrap values ≥70% are shown next to the relevant nodes. The tree is midpoint-rooted for purposes of clarity only.

Rotaviruses are nonenveloped viruses with a segmented genome consisting of 11 double-stranded RNA segments encoding six structural (VP1 to VP4, VP6 and VP7) and five to six nonstructural proteins (NSP1 to NSP6) [41], [42]. Rotavirus A is one of the three main species (the others being B and C) of the genus *Rotavirus,* from the family *Reoviridae*. There are several types of virus in this group, associated with a large number of diverse animal hosts [41], [43]. We identified sequences from pooled samples originating from specimen b8 (*Myotis mystacinus*) that gave significant hits matching sequences from this group of viruses. In BLASTx and BLASTn analyses, most of these sequences matched four of the 11 viral genome segments (Table 3). The presence of the VP1 and VP2 rotavirus related-sequences was confirmed in the lung tissue of specimen b8 by PCR with specific primers (Table 3, Fig. 4A and data not shown). Phylogenetic analysis based on the partial VP1 gene sequence demonstrated that this virus was more closely related to group A than other groups of rotaviruses (Fig. 4B). However, this sequence was found at a basal position in the phylogenetic tree, very distantly related to those clustering in this group, even the viral isolate recently identified in a single lesser horseshoe bat (*Rhinolophus hipposideros*) originated from China [44].

#### Identification of a new gammaretrovirus

**Figure 5:**
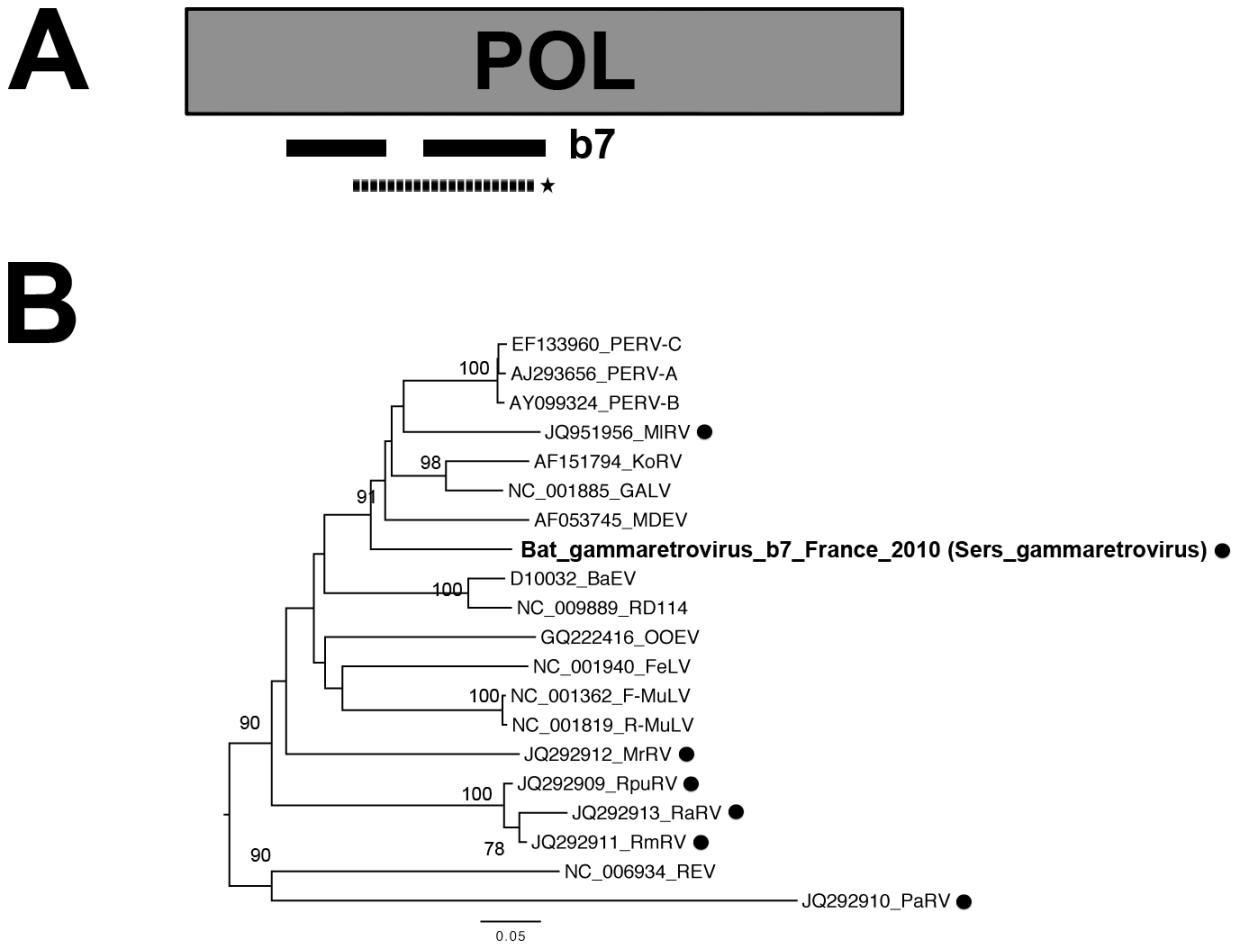
Phylogenetic analysis of the bat gammaretrovirus-related sequence. (A) Schematic representation of the partial genome structure encompassing the pol (almost 3,580 nt encoding, the polymerase of almost 1,190 aa) gene of the porcine endogenous retrovirus (GenBank number Y17013), with black bars corresponding to the longest contig sequences (>900 nt) of bat gammaretrovirus (named Sers gammaretrovirus) identified in samples from b7 (*Eptesicus serotinus)*. The genomic region amplified by PCR is represented by a dashed bar, and the sequence used for phylogenetic analysis is indicated with an asterisk. (B) Phylogenetic tree produced from the amino-acid alignment based on a selected region (155 aa) of the translated sequence obtained from the PCR product (almost 288 aa, approximate positions 173 to 423 of the pol protein of the porcine endogenous retrovirus). The bat gammaretrovirus-related sequence is indicated in bold, with circles in black indicating bat gammaretroviruses. The scale bar indicates branch length, and bootstrap values ≥70% are shown next to the relevant nodes. The tree is midpoint-rooted for purposes of clarity only. REV, reticuloendotheliosis virus; FeLV, feline leukemia virus; GALV, gibbon ape leukemia virus; F-MuLV, Friend MuLV; R-MuLV, Rauscher murine leukemia virus; M-MuLV, Moloney MuLV; M-CRV, murine type C retrovirus; PreXMRV-1/2, pre-xenotropic MuLV-related virus 1 and 2; PERV-A, porcine endogenous type C retrovirus class A; PERV-B, porcine endogenous retrovirus B; PERV-C, porcine endogenous retrovirus C; RD114, feline RD114 retrovirus; MDEV, *Mus dunni* endogenous virus; KoRV, koala retrovirus; OOEV, *Orcinus orca* endogenous retrovirus; BaEV, baboon endogenous virus; RpuRV, *Rhinolophus pusillus*; RmRV, *Rhinolophus megaphyllus*; RaRV, *Rhinolophus affinis* retrovirus; MrRV, *Myotis ricketti* retrovirus; PaRV, *Pteropus alecto* retrovirus and MlRV, *Megaderma lyra* retrovirus.

The genus *Gammaretrovirus* belongs to the subfamily *Orthoretrovirinae* in the family *Retroviridae*, and encompasses endogenous retroviruses with a simple genome organization [45]. These positive-sense enveloped viruses have an RNA genome encoding three proteins: gag (involved in viral protein synthesis), pol (corresponding to the protease, reverse transcriptase and integrase enzyme) and env (the viral glycoprotein), flanked by a long terminal repeat (LTR) at the 5′ and 3′ ends. Endogenous retroviruses correspond to integrated proviruses infecting germline cells and propagated by vertical transmission, occurring as either expressed or silent genomes (partially or completely defective) [46], [47]. In our study, several contig and read sequences with HSPs related to pol and env gammaretrovirus proteins were identified for specimen b7 (*Eptesicus serotinus)* (Table 3). The presence of gammaretroviral sequences was further confirmed in analyses of the lungs sample from this specimen, in PCR with specific primers based on the largest contig sequences matching the pol gene (Table 3 and Fig. 5A). Phylogenetic analysis performed on a selected region (155 aa) of the translated pol sequence obtained from the PCR product demonstrated that this virus, identified as Sers gammaretrovirus, belonged to the genus *Gammaretrovirus*, but differed from other members of this genus, including those recently identified in bats (Fig. 5B).

#### Identification of related borna disease viruses

**Figure 6:**
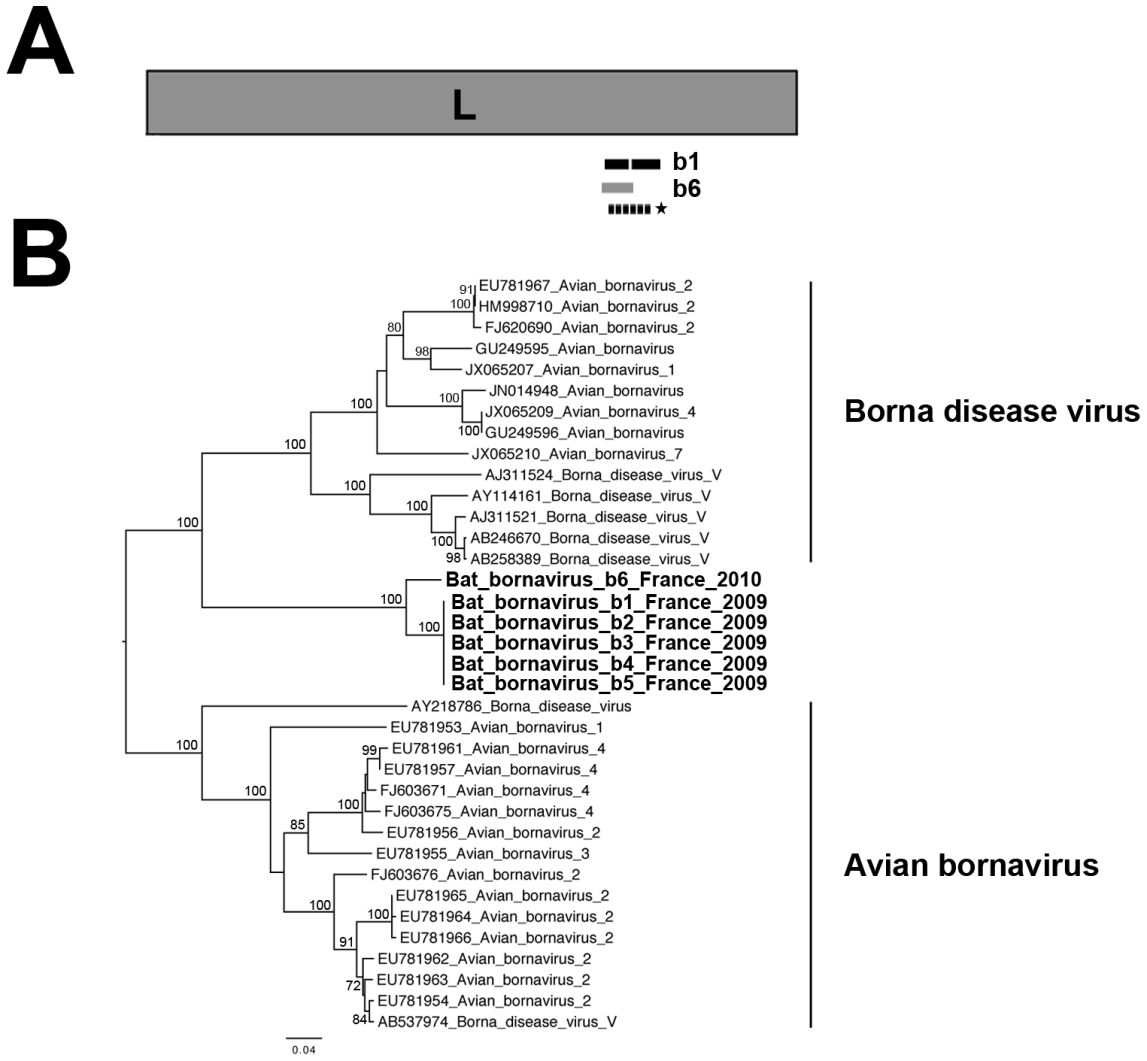
Phylogenetic analysis of the bat bornavirus-related sequences. (A) Schematic representation of the L gene (almost 5,140 nt encoding the RNA-dependent RNA polymerase of almost 1,710 aa) of the genome of Borna disease virus isolate bil (GenBank number ACG59353), with black and dark gray bars corresponding to the three bat bornavirus HSPs identified in samples b1 and b6, respectively. The position of the genome region amplified by PCR is represented by a dashed bar, and the sequence used for phylogenetic analysis is indicated with an asterisk. (B) Phylogenetic tree produced from the nucleotide alignment based on a selected region (214–216 nt, approximate position 3233–3446 of the L gene sequence of Borna disease virus isolate bil) of the sequence obtained from the PCR product. Bat bornavirus-related sequences are indicated in bold. The scale bar indicates branch length, and bootstrap values ≥70% are shown next to the relevant nodes. The tree is midpoint-rooted for purposes of clarity only.

*Borna disease virus* (BDV) is the prototype species of the family *Bornaviridae*, from the order Mononegavirales [48], [49]. A second phylogroup was recently identified in this family, with the discovery of avian bornavius (ABV) [50], [51]. Both phylogroups consist of enveloped viruses with a non segmented negative-stranded RNA (almost 8,900 nt long) encompassing the five canonical genes, encoding, in the following order, the nucleoprotein (N), the phosphoprotein (P), the matrix protein (M), the glycoprotein (G) and the polymerase (L) [52], [53]. Interestingly, endogenous sequences distantly related to some BDV genes (N, M and L) have been identified in the genomes of various mammals, including American microbats (*Myotis lucifugus*) [54], [55]. We identified HSPs derived from contig sequences closely related to bornavirus sequences in specimens b1 and b6 (Table 3). BLASTx analysis showed that all these sequences matched the same region of the viral polymerase, but that there was genetic variability between the two specimens (Fig. 6A). PCR on the brains of these specimens confirmed the presence of these sequences, but also showed them to be present in the brain or lungs samples of other bats (Table 3). With the exception of the bornavirus-related sequence from b6, all these sequences had premature stop codons at the beginning of the 5′ terminus, associated in one case with a 2 nt deletion with respect to the sequence from b6 (data not shown). Larger PCR products (>400 nt) were also obtained for the brains of specimens b3 and b5, the lungs of b6 and the liver of b9 (Table 3 and data not shown). After sequencing and BLASTx analysis, only the first 100 nt of these sequences were found to display identity to the L gene of bornavirus (no overlap with the previous 300 nt of sequence), the remaining nucleotide sequences matching eukaryotic sequences. Sequence alignment and phylogenetic analysis demonstrated that these bat bornavirus sequences were closely related and did not cluster with the two main viral phylogroups previously identified (Fig. 6B). Moreover, pairwise comparison (by BLASTp) of the translated b6 sequence (216 nt, 71 aa) and the sequence of the polymerase of an endogenous bornavirus found in the genome of *Myotis lucifugus* [54] showed these sequences to display 73% aa identity (data not shown). Overall, these observations are strongly suggestive of bat endogenous sequences.

#### Detection of other vertebrate viruses

In addition to the new viruses from the four main viral families described above, other read and small contig sequences matched sequences from many other vertebrate viruses, some with low nucleotide or amino-acid sequence similarities to known viruses.

For example, the pool of samples from the bat species *Myotis nattereri* (b6) contained several short read sequences (70 nt on average) related to the genus *Mastadenovirus* within the *Adenoviridae* family of double-stranded DNA viruses, with human adenovirus C identified as the closest relative (Table 3). High levels of amino-acid sequence similarity were observed for various adenoviral proteins, including the penton (E value = 0.0002, identity = 100%), penton base (E value = 0.0007, identity = 100%) and pIV (E value = 7e-09, identity = 94%) proteins. Two other sequences were found to be similar to sequences from recently described bat adenovirus isolates, with one contig sequence (114 nt, 84 aa) identified as related to bat adenovirus 2 in BLASTx analysis (E value = 4.7, identity = 47%), and a read sequence (76 nt) matching the bat adenovirus TJM in a BLASTn search (E value = 3e-17, identity = 92%) [56], [57].

Numerous read and contig sequences related to the family *Picobirnaviridae* (small double-stranded RNA viruses) were identified in five (specimens b1, b4, b5, b8 and b9) of the nine bat tissue sample pools (Table 3). These sequences displayed almost perfect nucleotide sequence identity to the gene encoding the RNA-dependent RNA polymerase of the human picobirnavirus, with high E values (ranging from 7e-5 to 1e-74).

#### Detection of invertebrate, plant or fungal viruses and bacteriophages

In addition to vertebrate viruses, the presence of viral sequences related to insect and plant viruses and bacteriophages has been reported for various bat samples, and some of these viruses had not previously been identified (see File S1).

## Discussion

We carried out a metagenomic analysis of the viral diversity found in insectivorous bats that had been in contact with humans in France. Pooled liver, lungs and brain (when available) samples from nine selected specimens from five different bat species were studied. Total RNA extraction following by sequence-independent PCR amplification process were realized, and high-throughput sequencing was performed with Illumina technology. By contrast to other previously published studies, no viral concentration step (centrifugation, filtration or nuclease use) was performed [16]–[18], [20]–[22]. Contig sequences generated by *de novo* assembly and unassembled reads were used for the taxonomic assignment of viruses to family level on the basis of BLASTx and BLASTn analyses. However, a large proportion of viral sequences could not be attributed to any known viral family. A large proportion of the identified contig and read sequences related to vertebrate viruses (mostly mammalian viruses), followed by invertebrate (mainly insect) and plant viruses, with a smaller proportion of sequences related to bacteriophages. The viromes of the different samples included numerous RNA virus families, as expected given that our metagenomic study was performed on total RNA extracts. Interestingly, we were also able to detect various DNA viruses (including phages and some eukaryotic viruses in particular), probably reflecting the presence of viral messenger RNA transcribed from the DNA genomes of these viruses, or even the presence of some traces of viral DNA after the extraction step.

It is difficult to compare our findings with other previously reported data for bat viromes, mostly due to differences in the species and the geographic origin of the bat specimens analyzed, and because most analyses were performed on guano samples (sometimes pooled with other types of samples, such as pharyngeal swabs for example) [16]–[18], [20] or focused only on vertebrate viruses [21]. However, as expected, the overall composition and proportion of the virome in bat organs differed considerably from the diversity of viruses reported for feces. The main difference concerned the number of sequences (contigs and reads) relating to vertebrate (mostly mammalian) viruses identified, ranging from 31% to 50% of all the viral sequences in our study, but below 10% in previous bat viromes based on guano [16]–[18], [20]. Our findings are closer to, but nonetheless still higher than those obtained in previous analyses of tissue samples (lungs), urine or pharyngeal swabs (28%) [21]. A recent report of the viral diversity found in pooled organs of bats from Myanmar exhibited some similar results when compared with our data, regarding the proportion of viral-related contig sequences (28%) or the distribution of viral families, especially those infecting vertebrates (Fig. 2) (data not shown) [22]. These results suggest that most of the mammalian viruses identified in our study do not reflect the environmental conditions (for example, the presence in guano of insect viruses may reflect the diet of the bat, and the presence of plant viruses in the guano may reflect the diet of the insects eaten) and are instead harbored by bat tissues. As we performed no viral concentration before RNA extraction and our deep sequencing approach based on Illumina technology is much more sensitive than some other methods [58], our study should reflect the most prevalent viruses present in each sample, thereby providing relevant information concerning the nature of the viral population colonizing bats. Based on the virome description obtained for each sample, we detected numerous viral contig and read sequences closely related to known viral sequences, and additional sequences potentially corresponding to new viruses.

The mammalian viruses identified included the first bat nairovirus, which was simultaneously detected in two different insectivorous bat species, *Myotis mystacinus* (specimen b8) and *Pipistrellus pipistrellus* (specimen b9), from the same family, *Vespertilionidae,* but collected from two different geographic locations in France. Phylogenetic analysis on the basis of a partial, conserved region of the polymerase sequence suggested that this virus belonged to a new species of the genus *Nairovirus*, distantly related to the seven species groups (or serogroups) of nairoviruses previously described. The presence of this species was confirmed molecularly, by specific PCR amplification, only in the lung tissue of these two bat specimens, raising questions about the route of dissemination and the physiopathology of infection in these animals. Nairoviruses are predominantly tick-borne, so it appears likely that this virus is also transmitted by these vectors [37], [38]. Indeed, bats are frequently parasitized by ticks, such as the argasid tick *Argas vespertilionis,* which has been shown to be subject to infection with various bacterial agents [59], [60]. The detection of this virus in two different bat species is also consistent with this hypothesis, highlighting its capacity to cross the species barrier and, potentially, to infect other mammalian hosts. Indeed, some species of the genus *Nairovirus* are known to be major human and/or animal pathogens, including Crimean Congo hemorrhagic fever virus, Nairobi sheep disease virus and Dugbe virus [37], [38]. The detection of this new virus adds to the list of bunyaviruses identified in bats, which now includes members of the genera *Orthobunyavirus*, *Hantavirus*, *Phlebovirus* and *Nairovirus* [1]. This further highlights the importance of viruses of the family *Bunyaviridae* as potential emerging zoonotic agents.

In addition to this new nairovirus, we also detected a bat rotavirus in the lungs of one microbat species, the whiskered bat, *Myotis mystacinus* (specimen b8). After deep sequencing, we identified contig and read sequences matching four (VP1, VP2, VP4 and VP7) of the 11 viral genome segments. These matches were confirmed by specific PCR for the VP1 and VP2 genes. Phylogenetic analysis based on the partial VP1 sequence, together with those obtained in BLAST analysis, demonstrated that this bat rotavirus was fairly related to group A, one of the 8 genotypes (A to H) identified to date within this genus [41], [61]. However, due to the availability of little or no sequence information available for the other genome segments, we cannot rule out the possibility of this virus being a reassortant. Full-length genome sequencing will be required to confirm its phylogenetic relationship and to determine the potential reassortment process. This is the second description of rotaviruses found in insectivorous bats, the first being a group A rotavirus recently identified in the gut of a single specimen of *Rhinolophus hipposideros* from Myanmar [44]. In addition, a reassortant group A rotavirus was also isolated from the feces of frugivorous *Eidolon helvum* bats in Kenya [62]. Unfortunately, sequence comparison with the VP1 segment of the latter was not possible due to the unavailability of the corresponding sequence. However, these results demonstrate that bats can harbor rotaviruses and extend previous findings of other bat viral isolates from the family *Reoviridae*, including viruses of the genus *Orthoreovirus* from fruit bats [1], [63] and, more recently, from microbats in Italy [64], and some other more dubious isolates for which a bat origin remains uncertain [65], [66]. Evidence for the interspecies transmission of rotaviruses and for genetic reassortment between animal species or between humans and animals has been reported, particularly in cases of close interactions between the species concerned [41], [43], [67], [68]. This highlights the need for better rotavirus surveillance in animals, particularly in wildlife, such as bats, for which few prevalence studies have been carried out. The detection of rotavirus sequences in lung tissue raises questions about the physiopathology of infection with this virus. Indeed, rotaviruses are known to be major enteric pathogens in humans and various other animal host species, including birds and mammals [69], [70], and they are generally thought to be restricted to the gastrointestinal tract. However, the detection of infection at extraintestinal locations, such as the lungs, has been reported in human cases and confirmed experimentally in animal models [71]–[73].

We report here the detection of sequences related to a new bat gammaretrovirus in lung tissue from the European bat species *Eptesicus serotinus* (specimen b7). Gammaretroviruses have previously been detected in only 10 other bat species, in China, Australia and, more recently, in Ghana [21], [74], [75]. Phylogenetic analysis on the partial amino-acid sequence of the pol protein indicated that this new gammaretrovirus was different from those previously described in bats, thus confirming that bats can harbor a number of genetically diverse gammaretrovirus species. It has recently been suggested that these animals may have played a major role as reservoir hosts during the diversification of mammalian gammaretroviruses [74], [75]. The contig sequences probably originated from viral transcripts, because total RNA was the starting biological material for our viral metagenomic analysis. However, we cannot exclude the possibility that we also detected the viral RNA genome of virions. Also and since DNA (viruses) were also detected, this novel gammaretrovirus could also be a endogeneous retrovirus. The detection of gammaretroviral sequences, by specific PCR amplification, in only one of the three tissue samples collected from specimen b7 is intriguing, but this may reflect higher levels of transcription in this tissue that in the other two tissues. However, the presence of retroviral sequences in lung samples is not surprising and confirms recent findings for African frugivorous bats (*Eidolon helvum*) [21].

Short sequences related to the L gene of bornavirus were identified in various tissue samples (brain, liver or lungs) collected from most of the bat specimens analyzed (b1–b6, b9). Surprisingly, all were identical or highly similar, and displayed high levels of amino-acid similarity to a bornavirus-related sequence recently discovered in the genome of *Myotis lucifugus* [54]. This suggests that these sequences may also be endogenous. It has been shown that similar endogenous bornavirus-related sequences can be expressed as mRNAs in other vertebrate models [55], [76]. Their presence and expression may confer some biological advantage on their hosts, such as natural resistance to the disease caused by the pathogen concerned [54]. This may account for the presence of these bornaviral sequences in all common pipistrelle specimens (*Pipistrellus pipistrellus*) tested and in two other bat species (*Hypsugo savii* and *Myotis nattereri*) of the five analyzed. However, screening for bornavirus-related sequences should be extended to other bat species.

We also detected diverse adenoviruses, probably including some new species, in the bat species *Myotis nattereri* (specimen b6). Various members of the genus *Mastadenovirus* have recently been shown to circulate widely in bats [16], [18], [20], [21], [77]. In particular, bat adenoviruses have been isolated from fecal samples of *Myotis* species in China and were detected in various tissues (including lungs and liver) collected from German bats belonging to the species *Pipistrellus pipistrellus* [56], [57]. Some of the reads and contig sequences of specimen b6 identified were found to be related to those of bat adenoviruses. Our results confirm that members of this virus family circulate widely in bats species, and that their potential spill-over transmission to other vertebrate species, including humans, merits further investigation.

Despite the limitations imposed by the small number and short length of some of the viral sequences, we detected human-related picobirnaviruses in three different bat species, *Pipistrellus pipistrellus* (specimens b1, b4 and b9), *Hypsugo savii* (specimen b5), and *Myotis mystacinus* (specimen b8). These results provide the evidence that picobirnarviruses are present in bats, confirming a previous report [22]. The members of the recently identified family *Picobirnarividae* are vertebrate viruses infecting a large spectrum of host species, including humans and diverse animals, such as other mammals, birds and reptiles [78]. These viruses have been found mostly in fecal samples and are, therefore, considered to be potential opportunistic enteric pathogens [78]–[81]. However, they have also been found in the respiratory tracts of humans and pigs [82], [83]. It is therefore not surprising that picobirnaviral sequences were detected in our pooled bat tissues (including lungs and liver). The prevalence of this viral family in the bat specimens analyzed appeared high (5/9 or 55%) and requires further confirmation in a larger panel of individuals and species. The high degree of sequence similarity between the bat picobirnaviruses found in our study and human picobirnaviruses raises questions about the transmission of these viruses and their capacity to spread to humans, confirming the zoonotic potential of this family of viruses [78], [84], [85].

In addition to these vertebrate viruses, we also identified new insect and plant viruses, mostly from viral families prevalent in the viromes obtained from bat guano, reflecting the diets of the insectivorous bats and of the insects they eat [16]–[18].

We developed and applied a simple, original methodology for the direct viral metagenomic analysis of complex tissue samples, such as brain, lungs or liver. Based on a high-throughput sequencing approach (Illumina technology), and the use of a dedicated bioinformatics workflow for dataset exploration, we generated preliminary data concerning the virome of pooled organs from French bats that had been in direct contact with humans. No close homolog of known human viral pathogens was detected in our study, but some of the new viruses described here should be investigated further to assess their zoonotic potential. Studies should be conducted, with the aim of isolating these viruses and evaluating their capacity to replicate *in vitro* in cell lines from different species, including human cell lines. Screening for the presence of these new mammalian viruses, by PCR with specific or degenerate primers, in a large panel of bat specimens and additional biological samples, such as saliva, urine or feces, would be also informative as it would allow us to evaluate the prevalence of these viruses, their mode of spread and infection, and their capacity to circulate in various host species. Additional investigations are also required to find a causal link between disease in bats and the viruses identified.

As in other bat virome studies, a large number of sequences remained unclassified after taxonomic analysis on the basis of BLAST analysis, resulting in an underestimation of the total diversity of the viruses found in these animals [16]–[18], [20], [22]. However, we provide some comprehensive clues to the nature of the viral communities present in the most common French bat species. In particular, we show that viral diversity is high within a given bat species, as in *Pipistrellus pipistrellus* specimens b1–b4 and b9, but also between different species, thus confirming previous results [16], also we can not exclude that the observed differences in viral composition might also be related to differences in storage time and temperature between bats before necropsy was performed. We demonstrated that a single bat specimen could harbor multiple potentially zoonotic viruses, such as Ahun nairovirus and Maule rotavirus in specimen b8 (*Myotis mystacinus*). Further studies are required in a larger number of specimens from the same bat species and from additional, as yet unanalyzed species. In addition, a comparison of the viromes of bat specimens from other sites in Europe should extend the description of the diversity of viruses circulating in European bat populations.

## Supporting Information

Figure S1 **Phylogenetic analysis of the bat dicistrovirus-related sequence.** (A) Schematic representation of the ORF1 gene (almost 5,330 nt encoding the nonstructural protein precursor of almost 1,780 aa) from the Himetobi P virus (GenBank number AB183472), with the black bar corresponding to the longest contig sequences (>1,800 nt) of bat dicistrovirus (named Paris dicistrovirus) identified in specimen b2 (Pipistrellus pipistrellus). The position of the genome region amplified by PCR is represented by a dashed bar, and the sequence used for phylogenetic analysis is indicated with an asterisk. (B) Phylogenetic tree produced from the amino-acid alignment based on the partial ORF1 sequence (606 aa, approximate aa positions 1097 to 1702 of the nonstructural protein precursor of Himetobi P virus). The bat dicistrovirus-related sequence is indicated in bold, within the various viral genera. The scale bar indicates branch length, and bootstrap values ≥70% are shown next to the relevant nodes. The tree is midpoint-rooted for purposes of clarity only.
(PDF)

Figure S2 **Phylogenetic analysis of the bat nodavirus-related sequence.** (A) Schematic representation of the RNA-1 genome segment (almost 3,110 nt, encoding the RNA-dependent RNA polymerase or ORF1a of almost 1,000 aa, in addition to ORF1b or protein B2, of almost 105 aa) corresponding to protein A of Boolarra virus (GenBank number NC_004142), with the black bar corresponding to the longest contig sequence (>1,500 nt) of the bat nodavirus (named Sers nodavirus) identified in specimen b7 (Eptesicus serotinus). The position of the genome region amplified by PCR is represented by a dashed bar, and the sequence used for phylogenetic analysis is indicated with an asterisk. (B) Phylogenetic tree produced from the amino-acid alignment based on the partial ORF1a sequence (531 aa, approximate aa positions 7 to 537 of the protein A of Boolarra virus) translated from the longest contig. The bat nodavirus-related sequence is indicated in bold, within the various viral genera. The scale bar indicates branch length, and bootstrap values ≥70% are shown next to the relevant nodes. The tree is midpoint-rooted for purposes of clarity only.
(PDF)

Figure S3 **Phylogenetic analysis of the bat luteovirus-related sequence.** (A) Schematic representation of the ORF1-2 genome region (almost 3,150 nt) encoding the RNA-dependent RNA polymerase as a P1–P2 fusion protein (almost 1050 aa) from the pea enation mosaic virus-1 (Uniprot number P29154), with the black bar corresponding to the longest HSP sequence (>700 nt) from bat luteovirus (named Bordeaux luteovirus) identified in samples from b3 (Pipistrellus pipistrellus). (B) Phylogenetic tree produced from the amino-acid alignment based on the partial ORF1-2 sequence (203 aa, approximate aa positions 698 to 907 of the pea enation mosaic virus-1 protein P1–P2) translated from the longest HSP. The bat luteovirus-related sequence is indicated in bold, within the various viral genera. The scale bar indicates branch length, and bootstrap values ≥70% are shown next to the relevant nodes. The tree is midpoint-rooted for purposes of clarity only.
(PDF)

Figure S4 **Phylogenetic analysis of the bat sobemovirus-related sequence.** (A) Schematic representation of the ORF4 gene (almost 710 nt encoding the coat protein of almost 235 aa) of the Sowbane mosaic virus, (GenBank number NC_011187), with black bars corresponding to the longest contig sequence (>670 nt) from bat sobemovirus (named Bordeaux sobemovirus) identified in samples from b3 (Pipistrellus pipistrellus). (B) Phylogenetic analysis based on the partial coat protein amino-acid sequence (226 aa, approximate aa positions 1 to 237 of the capsid protein of Sowbane mosaic virus) translated from the contig of sample b3. (B) Phylogenetic tree produced from the amino-acid alignment based on the partial ORF4 sequence (206 aa) translated from the longest contig. The bat sobemovirus-related sequence is indicated in bold, within the various viral genera. The scale bar indicates branch length, and bootstrap values ≥70% are shown next to the relevant nodes. The tree is midpoint-rooted for purposes of clarity only.
(PDF)

Table S1 **Identification and distribution of sequences of insect and plant viruses of interest among the various bat specimens and tissue samples analyzed.**
(PDF)

File S1 **Identification of new insect and plant viruses.**
(PDF)
