# Supplementary material for: A Preliminary Study of Viral Metagenomics of French Bat Species in Contact with Humans: Identification of New Mammalian Viruses
Source: PLoS One. 2014 Jan 29;9(1):e87194. doi: 10.1371/journal.pone.0087194 (PMC3906132; doi:10.1371/journal.pone.0087194)
Supplement: Figure S1 — Phylogenetic analysis of the bat dicistrovirus-related sequence. (A) Schematic representation of the ORF1 gene (almost 5,330 nt encoding the nonstructural protein precursor of almost 1,780 aa) from the Himetobi P virus (GenBank number AB183472), with the black bar corresponding to the longest contig sequences (>1,800 nt) of bat dicistrovirus (named Paris dicistrovirus) identified in specimen b2 (Pipistrellus pipistrellus). The position of the genome region amplified by PCR is represented by a dashed bar, and the sequence used for phylogenetic analysis is indicated with an asterisk. (B) Phylogenetic tree produced from the amino-acid alignment based on the partial ORF1 sequence (606 aa, approximate aa positions 1097 to 1702 of the nonstructural protein precursor of Himetobi P virus). The bat dicistrovirus-related sequence is indicated in bold, within the various viral genera. The scale bar indicates branch length, and bootstrap values ≥70% are shown next to the relevant nodes. The tree is midpoint-rooted for purposes of clarity only. (PDF) [file pone.0087194.s001.pdf]

Figure S1

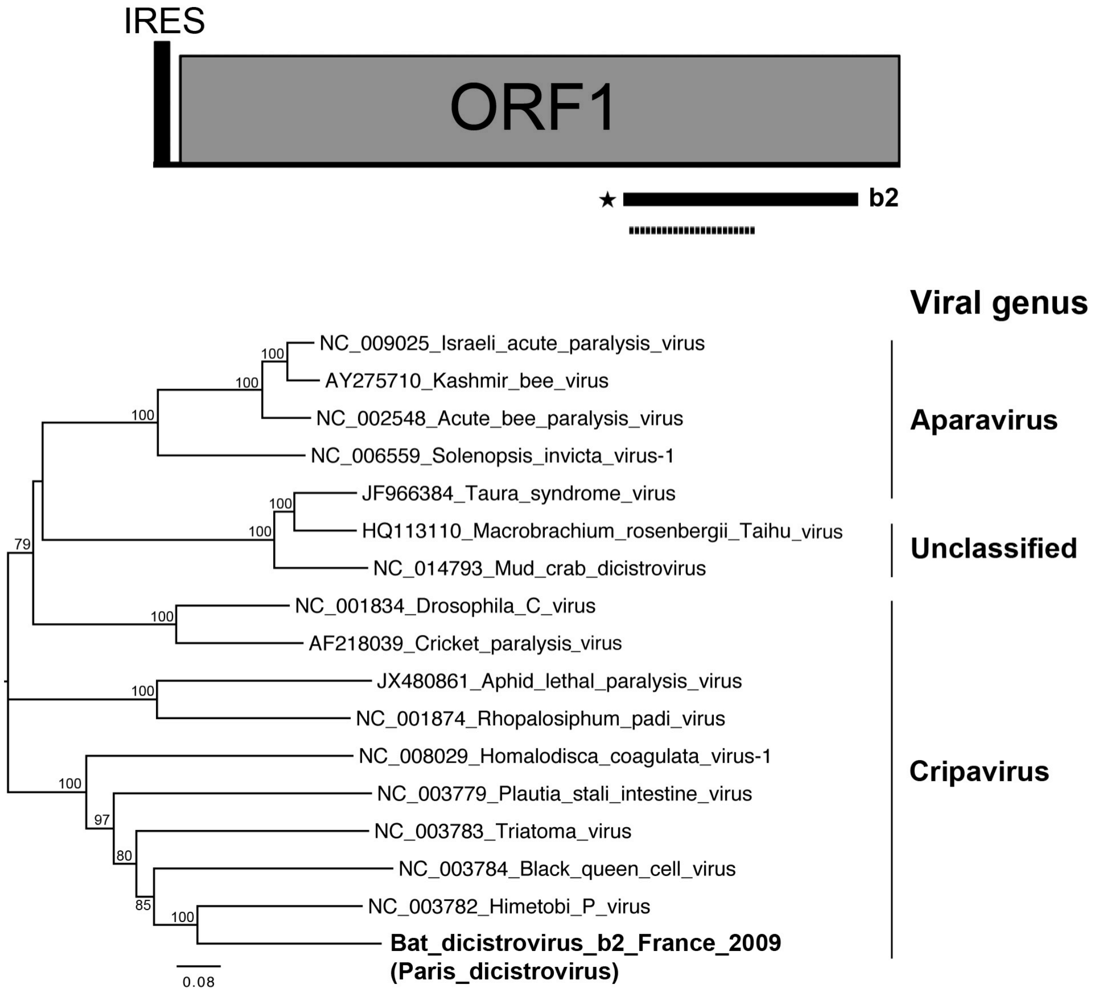

Phylogenetic analysis of the bat dicistrovirus-related sequence. (A) Schematic representation of the ORF1 gene (almost 5,330 nt encoding the nonstructural protein precursor of almost 1,780 aa) from the Himetobi P virus (GenBank number AB183472), with the black bar corresponding to the longest contig sequences (>1,800 nt) of bat dicistrovirus (named Paris dicistrovirus) identified in specimen b2 (*Pipistrellus pipistrellus*). The position of the genome region amplified by PCR is represented by a dashed bar, and the sequence used for phylogenetic analysis is indicated with an asterisk. (B) Phylogenetic tree produced from the amino-acid alignment based on the partial ORF1 sequence (606 aa, approximate aa positions 1097 to 1702 of the nonstructural protein precursor of Himetobi P virus). The bat dicistrovirus-related sequence is indicated in bold, within the various viral genera. The scale bar indicates branch length, and bootstrap values ≥ 70% are shown next to the relevant nodes. The tree is midpoint-rooted for purposes of clarity only.
