# Supplementary material for: A Preliminary Study of Viral Metagenomics of French Bat Species in Contact with Humans: Identification of New Mammalian Viruses
Source: PLoS One. 2014 Jan 29;9(1):e87194. doi: 10.1371/journal.pone.0087194 (PMC3906132; doi:10.1371/journal.pone.0087194)
Supplement: Figure S2 — Phylogenetic analysis of the bat nodavirus-related sequence. (A) Schematic representation of the RNA-1 genome segment (almost 3,110 nt, encoding the RNA-dependent RNA polymerase or ORF1a of almost 1,000 aa, in addition to ORF1b or protein B2, of almost 105 aa) corresponding to protein A of Boolarra virus (GenBank number NC_004142), with the black bar corresponding to the longest contig sequence (>1,500 nt) of the bat nodavirus (named Sers nodavirus) identified in specimen b7 (Eptesicus serotinus). The position of the genome region amplified by PCR is represented by a dashed bar, and the sequence used for phylogenetic analysis is indicated with an asterisk. (B) Phylogenetic tree produced from the amino-acid alignment based on the partial ORF1a sequence (531 aa, approximate aa positions 7 to 537 of the protein A of Boolarra virus) translated from the longest contig. The bat nodavirus-related sequence is indicated in bold, within the various viral genera. The scale bar indicates branch length, and bootstrap values ≥70% are shown next to the relevant nodes. The tree is midpoint-rooted for purposes of clarity only. (PDF) [file pone.0087194.s002.pdf]

Figure S2

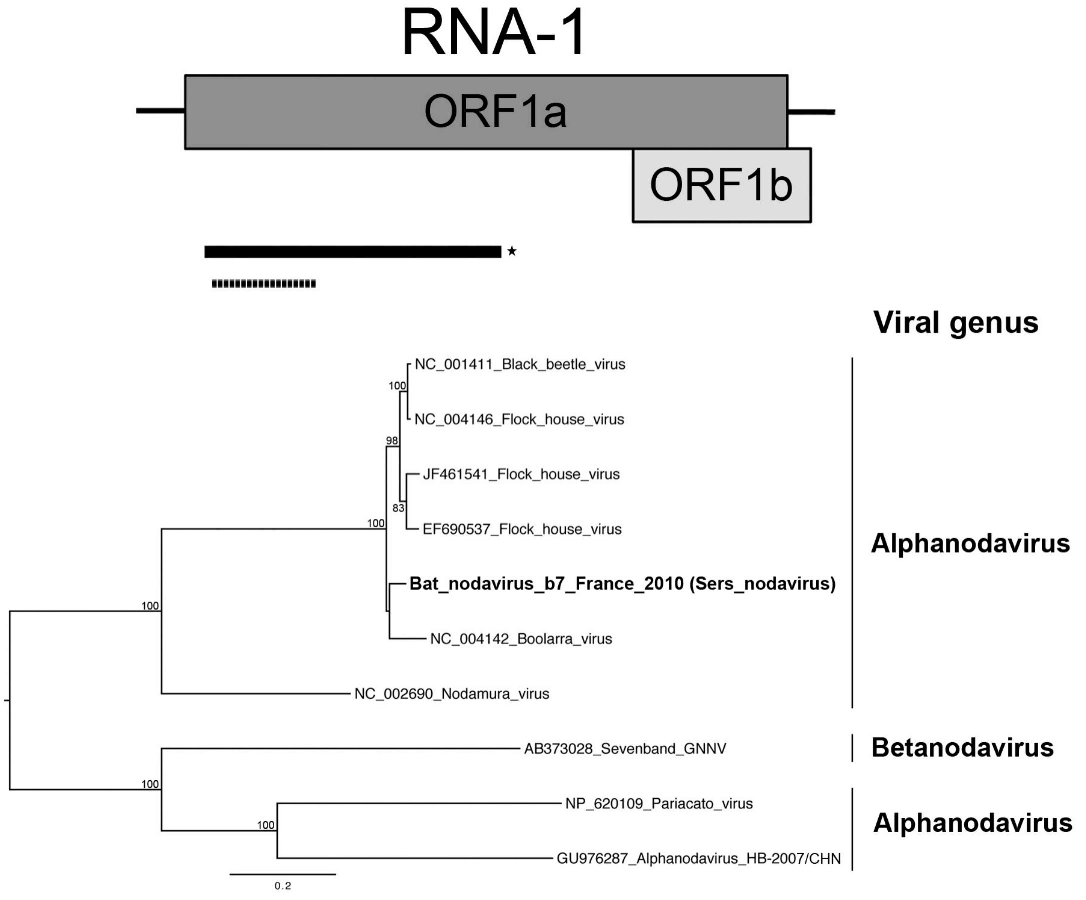

Phylogenetic analysis of the bat nodavirus-related sequence. (A) Schematic representation of the RNA-1 genome segment (almost 3,110 nt, encoding the RNA-dependent RNA polymerase or ORF1a of almost 1,000 aa, in addition to ORF1b or protein B2, of almost 105 aa) corresponding to protein A of Boolarra virus (GenBank number NC_004142), with the black bar corresponding to the longest contig sequence (>1,500 nt) of the bat nodavirus (named Sers nodavirus) identified in specimen b7 (*Eptesicus serotinus*). The position of the genome region amplified by PCR is represented by a dashed bar, and the sequence used for phylogenetic analysis is indicated with an asterisk. (B) Phylogenetic tree produced from the amino-acid alignment based on the partial ORF1a sequence (531 aa, approximate aa positions 7 to 537 of the protein A of Boolarra virus) translated from the longest contig. The bat nodavirus-related sequence is indicated in bold, within the various viral genera. The scale bar indicates branch length, and bootstrap values ≥ 70% are shown next to the relevant nodes. The tree is midpoint-rooted for purposes of clarity only.
