# Supplementary material for: A Preliminary Study of Viral Metagenomics of French Bat Species in Contact with Humans: Identification of New Mammalian Viruses
Source: PLoS One. 2014 Jan 29;9(1):e87194. doi: 10.1371/journal.pone.0087194 (PMC3906132; doi:10.1371/journal.pone.0087194)
Supplement: Figure S3 — Phylogenetic analysis of the bat luteovirus-related sequence. (A) Schematic representation of the ORF1-2 genome region (almost 3,150 nt) encoding the RNA-dependent RNA polymerase as a P1–P2 fusion protein (almost 1050 aa) from the pea enation mosaic virus-1 (Uniprot number P29154), with the black bar corresponding to the longest HSP sequence (>700 nt) from bat luteovirus (named Bordeaux luteovirus) identified in samples from b3 (Pipistrellus pipistrellus). (B) Phylogenetic tree produced from the amino-acid alignment based on the partial ORF1-2 sequence (203 aa, approximate aa positions 698 to 907 of the pea enation mosaic virus-1 protein P1–P2) translated from the longest HSP. The bat luteovirus-related sequence is indicated in bold, within the various viral genera. The scale bar indicates branch length, and bootstrap values ≥70% are shown next to the relevant nodes. The tree is midpoint-rooted for purposes of clarity only. (PDF) [file pone.0087194.s003.pdf]

Figure S3

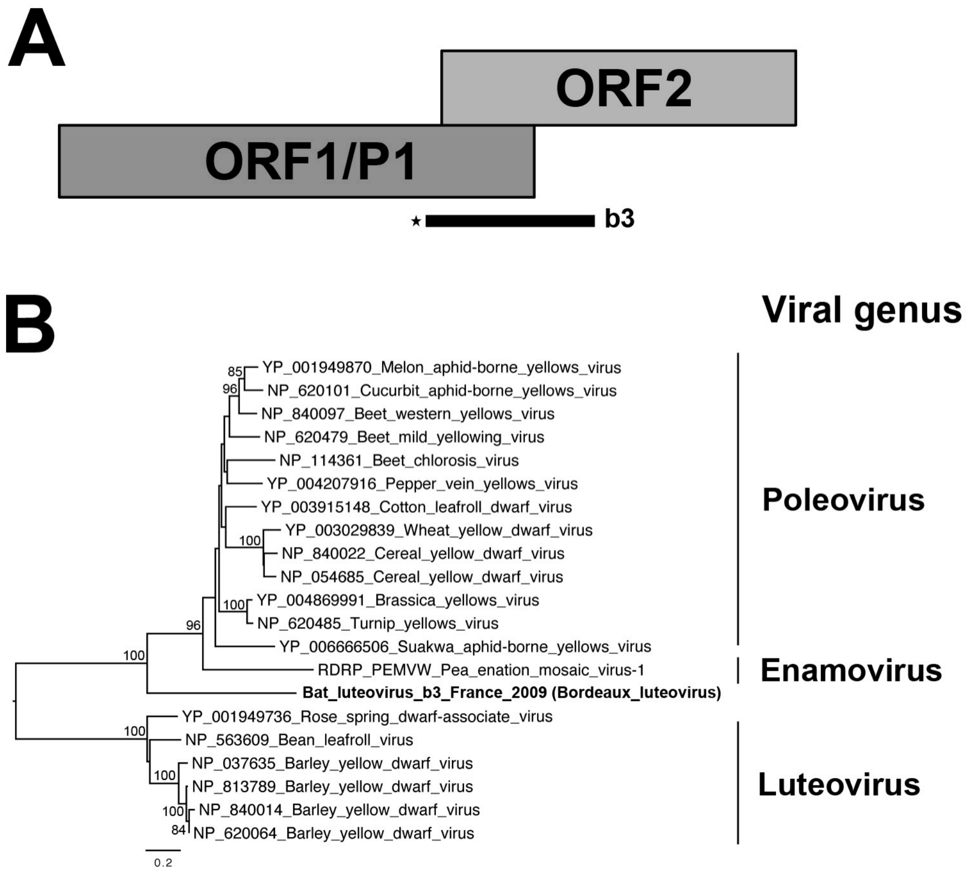

Phylogenetic analysis of the bat luteovirus-related sequence. (A) Schematic representation of the ORF1-2 genome region (almost 3,150 nt) encoding the RNA-dependent RNA polymerase as a P1-P2 fusion protein (almost 1050 aa) from the pea enation mosaic virus-1 (Uniprot number P29154), with the black bar corresponding to the longest HSP sequence (>700 nt) from bat luteovirus (named Bordeaux luteovirus) identified in samples from b3 (*Pipistrellus pipistrellus*). (B) Phylogenetic tree produced from the amino-acid alignment based on the partial ORF1-2 sequence (203 aa, approximate aa positions 698 to 907 of the pea enation mosaic virus-1 protein P1-P2) translated from the longest HSP. The bat luteovirus-related sequence is indicated in bold, within the various viral genera. The scale bar indicates branch length, and bootstrap values ≥ 70% are shown next to the relevant nodes. The tree is midpoint-rooted for purposes of clarity only.
