# Supplementary material for: A Preliminary Study of Viral Metagenomics of French Bat Species in Contact with Humans: Identification of New Mammalian Viruses
Source: PLoS One. 2014 Jan 29;9(1):e87194. doi: 10.1371/journal.pone.0087194 (PMC3906132; doi:10.1371/journal.pone.0087194)
Supplement: Figure S4 — Phylogenetic analysis of the bat sobemovirus-related sequence. (A) Schematic representation of the ORF4 gene (almost 710 nt encoding the coat protein of almost 235 aa) of the Sowbane mosaic virus, (GenBank number NC_011187), with black bars corresponding to the longest contig sequence (>670 nt) from bat sobemovirus (named Bordeaux sobemovirus) identified in samples from b3 (Pipistrellus pipistrellus). (B) Phylogenetic analysis based on the partial coat protein amino-acid sequence (226 aa, approximate aa positions 1 to 237 of the capsid protein of Sowbane mosaic virus) translated from the contig of sample b3. (B) Phylogenetic tree produced from the amino-acid alignment based on the partial ORF4 sequence (206 aa) translated from the longest contig. The bat sobemovirus-related sequence is indicated in bold, within the various viral genera. The scale bar indicates branch length, and bootstrap values ≥70% are shown next to the relevant nodes. The tree is midpoint-rooted for purposes of clarity only. (PDF) [file pone.0087194.s004.pdf]

Figure S4

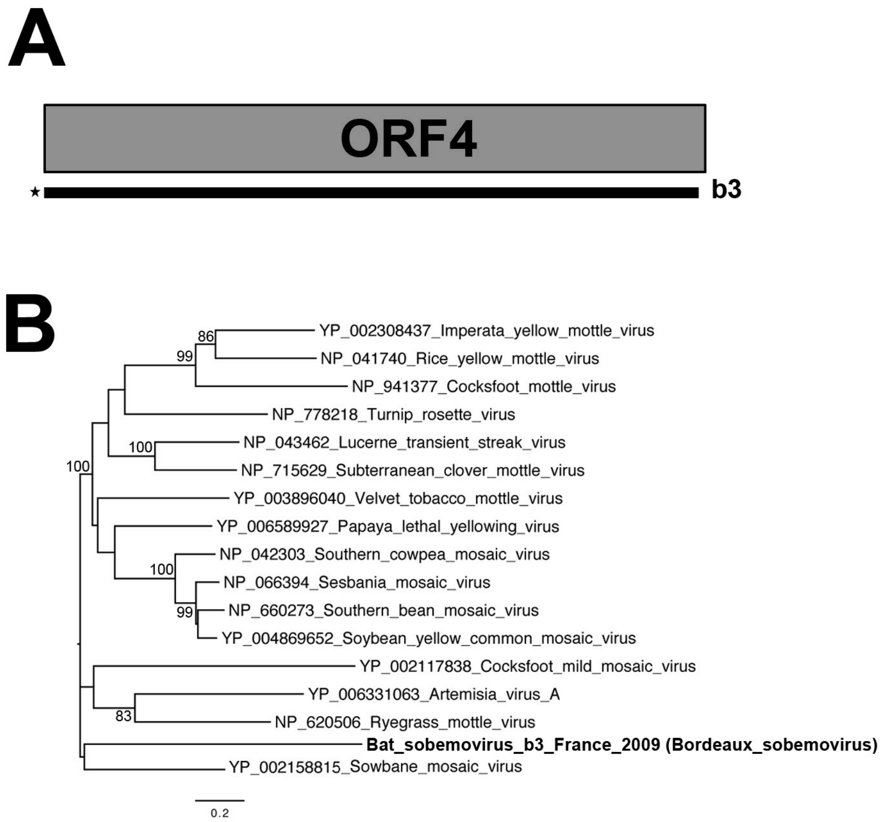

Phylogenetic analysis of the bat sobemovirus-related sequence. (A) Schematic representation of the ORF4 gene (almost 710 nt encoding the coat protein of almost 235 aa) of the Sowbane mosaic virus, (GenBank number NC_011187), with black bars corresponding to the longest contig sequence (> 670 nt) from bat sobemovirus (named Bordeaux sobemovirus) identified in samples from b3 (*Pipistrellus pipistrellus*). (B) Phylogenetic analysis based on the partial coat protein amino-acid sequence (226 aa, approximate aa positions 1 to 237 of the capsid protein of Sowbane mosaic virus) translated from the contig of sample b3. (B) Phylogenetic tree produced from the amino-acid alignment based on the partial ORF4 sequence (206 aa) translated from the longest contig. The bat sobemovirus-related sequence is indicated in bold, within the various viral genera. The scale bar indicates branch length, and bootstrap values ≥ 70% are shown next to the relevant nodes. The tree is midpoint-rooted for purposes of clarity only.
