# Supplementary material for: A Preliminary Study of Viral Metagenomics of French Bat Species in Contact with Humans: Identification of New Mammalian Viruses
Source: PLoS One. 2014 Jan 29;9(1):e87194. doi: 10.1371/journal.pone.0087194 (PMC3906132; doi:10.1371/journal.pone.0087194)
Supplement: Table S1 — Identification and distribution of sequences of insect and plant viruses of interest among the various bat specimens and tissue samples analyzed. (PDF) [file pone.0087194.s005.pdf]

Table S1 : Identification and distribution of sequences of insect and plant viruses of interest among the various bat specimens and tissue samples analyzed

		Bat species, specimens and tissue samples[a]																								
Virus	Sequence detection[b]	*Pipistrellus pipistrellus*															*Hypsugo savii*			*Myotis nattereri*			*Eptesicus serotinus*			*Myotis mystacinus*	
		b1			b2			b3			b4			b9			b5			b6			b7			b8	
		Br	Li	Lu	Br	Li	Lu	Br	Li	Lu	Br	Li	Lu	Li	Lu	Br	Li	Lu	Br	Li	Lu	Br	Li	Lu	Li	Lu
Dicistrovirus	HTS	- [c] (tissue pool)			+[c] (tissue pool) *[ORF1-2]*			- (tissue pool)			- (tissue pool)			- (tissue pool)		- (tissue pool)			- (tissue pool)			- (tissue pool)			- (tissue pool)	
	PCR/Sanger	-	-	-	-	-	+ *[ORF1]*	-	-	-	-	-	-	-	-	-	-	-	-	-	-	-	-	-	-	-
Nodavirus	HTS	- (tissue pool)			- (tissue pool)			- (tissue pool)			- (tissue pool)			- (tissue pool)		- (tissue pool)			- (tissue pool)			+ (tissue pool) *[multiple genes]*			- (tissue pool)	
	PCR/Sanger	-	-	-	-	-	-	-	-	-	-	-	-	-	-	-	-	-	-	-	-	+ *[ORF1a]*	-	-	-	-
Luteovirus	HTS	- (tissue pool)			- (tissue pool)			+ (tissue pool) *[ORF1-2]*			- (tissue pool)			- (tissue pool)		- (tissue pool)			- (tissue pool)			- (tissue pool)			- (tissue pool)	
	PCR/Sanger	ND[d]	ND	ND	ND	ND	ND	ND	ND	ND	ND	ND	ND	ND	ND	ND	ND	ND	ND	ND	ND	ND	ND	ND	ND	ND
Sobemovirus	HTS	- (tissue pool)			- (tissue pool)			+ (tissue pool) *[ORF4]*			- (tissue pool)			- (tissue pool)		- (tissue pool)			- (tissue pool)			- (tissue pool)			- (tissue pool)	
	PCR/Sanger	ND	ND	ND	ND	ND	ND	ND	ND	ND	ND	ND	ND	ND	ND	ND	ND	ND	ND	ND	ND	ND	ND	ND	ND	ND

Legend :

[a] : Br = brain, Li = liver, Lu = lungs

[b] : Matching (HTS) or targeting (PCR/Sanger) viral genes (in brackets), with ORF = open reading frame

[c] : + = positive, - = negative

[d] : ND = not done
