# Supplementary material for: A Preliminary Study of Viral Metagenomics of French Bat Species in Contact with Humans: Identification of New Mammalian Viruses
Source: PLoS One. 2014 Jan 29;9(1):e87194. doi: 10.1371/journal.pone.0087194 (PMC3906132; doi:10.1371/journal.pone.0087194)
Supplement: File S1 — Identification of new insect and plant viruses. (PDF) [file pone.0087194.s006.pdf]

Supporting Information

Identification of new insect and plant viruses

The virome of insectivorous bat feces has been shown to consist largely of insect viruses, followed by plant viruses, reflecting the insect-based diet of these bats, and plant-based diet of these insects [1-3]. Conversely, our study, based on the analysis of tissue samples, led to the detection of only a few new insect viruses, and even fewer new plant viruses.

Identification of new insect viruses

Based on BLASTx and BLASTn analyses, numerous read and contig sequences from pooled bat samples were identified as taxonomically related to families of viruses infecting invertebrates, mostly the *Polydnaviridae*, *Baculoviridae*, *Dicistroviridae* and *Nodaviridae*. For the last two of these families, we were able to identify and confirm the presence of putative new insect viruses, in bat specimens b2 (*Pipistrellus pipistrellus*) and b7 (*Eptesicus serotinus*), respectively.

The family *Dicistroviridae* encompasses small single-stranded positive-sense RNA viruses infecting invertebrates, which can be distinguished from members of the "picornavirus-like superfamily" taxon on the basis of the location of the non-structural protein genes (ORF-1) at the 5' end of the genome rather than at the 3' end. Dicistroviruses have been isolated from six invertebrate orders from the Insecta and decapod crustaceans [4]. In this study, multiple contig sequences from bat specimen b2 (*Pipistrellus pipistrellus*) displayed various HSPs related to the family *Dicistroviridae*, matching structural and nonstructural viral proteins in BLASTx analysis (Table S1). The low degree of nucleotide sequence identity observed, with overlapping regions for some of these HSP sequences, suggested that different dicistroviruses were present (data not shown). Based on the longest HSP sequence (1,819 nt) matching the ORF1 gene, a specific set of primers was designed and used to confirm the presence of dicistrovirus sequences in the lungs of specimen b2 (Table S1 and Fig. S1A). Phylogenetic analysis demonstrated that the related virus, Paris dicistrovirus, most closely matched the

nonstructural polyprotein region of the *Cripavirus* genus. However, it was different from the other dicistroviruses described (Fig. S1B).

The family *Nodaviridae* encompasses non enveloped isometric viruses with two positive sense-strand RNA molecules, RNA-1 and RNA-2. The RNA-1 segment encodes protein A (ORF1a), a catalytic subunit of the RNA-dependent RNA polymerase (RdRp), and protein B (ORF1b), whereas the second segment, RNA-2, encodes the coat precursor protein alpha [5]. This family contains two genera, *Alphanodavirus* and *Betanodavirus*, which principally infect insects and fish [6]. We identified multiple read and contig sequences from pooled tissues of bat specimen b7 (*Eptesicus serotinus*) that matched nodavirus sequences in BLASTn and BLASTx analysis (Table S1). The presence of nodavirus sequences was further confirmed by PCR on the brain sample of this specimen (Table S1 and Fig. S2A). Phylogenetic analysis performed after translation to obtain the amino-acid sequence encoded by this contig demonstrated that this virus, the Sers nodavirus, belonged to the genus *Alphanodavirus*, and was closely related to Boolarra virus (Fig. S2B).

Identification of new plant viruses

As previously indicated, multiple read and contig sequences from pooled samples from bats matched sequences from various families of viruses infecting plants, such as *Phycodnaviridae*, *Tombusviridae* and *Luteoviridae*, in BLASTx and, to a lesser extent, BLASTn analysis. Most of these sequences displayed low levels of sequence identity to known viral proteins, suggesting the presence of new viral species. For example, in specimen b3 (*Pipistrellus pipistrellus*), we identified a putative new viral genus from *Luteoviridae* and a different viral species from the genus *Sobemovirus*.

Luteoviruses are simple non enveloped, icosahedral viruses with one single-stranded, positive-sense RNA of about 6 kb in size encompassing several ORFs. ORF1-2 encodes the RNA-dependent RNA polymerase (produced as a P1-P2 fusion protein). Luteoviruses are aphid-borne viruses that infect a diverse range of plants, including crop plants of interest to humans and animals. Specimen b3 yielded one contig sequence matching sequence in the luteovirus genome (Table S1 and Fig. S3A).

Phylogenetic analysis, based on the translated HSP, confirmed that this virus, tentatively named Bordeaux luteovirus, belonged to the family *Luteoviridae*, and demonstrated that it was a putative new viral genus closely related to the genus *Enamovirus* (Fig. S3B).

The genus *Sobemovirus* encompasses plant RNA viruses with icosahedral virions containing a positive-sense single-stranded RNA genome of almost 4 kb in length containing three or four ORFs encoding various proteins, including the coat protein (ORF4). The host range of each sobemovirus species is narrow and confined to a few plant species from *Poaceae* or *Fabaceae*. In this study, we identified one HSP derived from a single contig sequence from the same bat specimen b3, which was found to be closely related to the coat protein of sobemoviruses in BLASTx analysis (Table S1 and Fig. S4A). Phylogenetic analysis indicated that this virus that we named Bordeaux sobemovirus belonged to the genus *Sobemovirus* and represented a new, distinctive viral species.

Identification of bacteriophage sequences

Three main families of phages, *Myoviridae*, *Siphoviridae* and *Podoviridae,* were represented in our bat virome analysis, accounting for almost 8% and 10% the total number of viral contigs and reads, respectively.

Phage-related sequences corresponded mostly to enterobacterial phages, which probably infected the intestinal bacterial population. However, the length of contig sequences obtained after *de novo* assembly was not sufficient for subsequent relevant sequence comparisons or phylogenetic analysis.

References

1. Donaldson EF, Haskew AN, Gates JE, Huynh J, Moore CJ, et al. (2010) Metagenomic analysis of the viromes of three North American bat species: viral diversity among different bat species that share a common habitat. J Virol 84: 13004-13018.
2. Ge X, Li Y, Yang X, Zhang H, Zhou P, et al. (2012) Metagenomic analysis of viruses from bat fecal samples reveals many novel viruses in insectivorous bats in China. J Virol 86: 4620-4630.
3. Li L, Victoria JG, Wang C, Jones M, Fellers GM, et al. (2010) Bat guano virome: predominance of dietary viruses from insects and plants plus novel mammalian viruses. J Virol 84: 6955-6965.
4. Bonning BC, Miller WA (2010) Dicistroviruses. Annu Rev Entomol 55: 129-150.

5. Liu C, Zhang J, Yi F, Wang J, Wang X, et al. (2006) Isolation and RNA1 nucleotide sequence determination of a new insect nodavirus from Pieris rapae larvae in Wuhan city, China. Virus Res 120: 28-35.
6. Bai H, Wang Y, Li X, Mao H, Li Y, et al. (2011) Isolation and characterization of a novel alphanodavirus. Virol J 8: 311.
